# Mapping neurotransmitter systems to the structural and functional organization of the human neocortex

**DOI:** 10.1038/s41593-022-01186-3

**Published:** 2022-10-27

**Authors:** Justine Y. Hansen, Golia Shafiei, Ross D. Markello, Kelly Smart, Sylvia M. L. Cox, Martin Nørgaard, Vincent Beliveau, Yanjun Wu, Jean-Dominique Gallezot, Étienne Aumont, Stijn Servaes, Stephanie G. Scala, Jonathan M. DuBois, Gabriel Wainstein, Gleb Bezgin, Thomas Funck, Taylor W. Schmitz, R. Nathan Spreng, Marian Galovic, Matthias J. Koepp, John S. Duncan, Jonathan P. Coles, Tim D. Fryer, Franklin I. Aigbirhio, Colm J. McGinnity, Alexander Hammers, Jean-Paul Soucy, Sylvain Baillet, Synthia Guimond, Jarmo Hietala, Marc-André Bedard, Marco Leyton, Eliane Kobayashi, Pedro Rosa-Neto, Melanie Ganz, Gitte M. Knudsen, Nicola Palomero-Gallagher, James M. Shine, Richard E. Carson, Lauri Tuominen, Alain Dagher, Bratislav Misic

**Affiliations:** 1 Montréal Neurological Institute, McGill University, Montréal, QC Canada; 2 Yale PET Center, Yale School of Medicine, New Haven, CT USA; 3 Department of Radiology and Biomedical Imaging, Yale School of Medicine, New Haven, CT USA; 4 Department of Psychiatry, McGill University, Montréal, QC Canada; 5 Department of Psychology, Center for Reproducible Neuroscience, Stanford University, Stanford, CA USA; 6 Neurobiology Research Unit, Cimbi & OpenNeuroPET, Copenhagen University Hospital Rigshospitalet, Copenhagen, Denmark; 7 Department of Neurology, Medical University of Innsbruck, Innsbruck, Austria; 8 Cognitive Pharmacology Research Unit, UQAM, Montréal, QC Canada; 9 McGill University Research Centre for Studies in Aging, Douglas Hospital, McGill University, Montréal, QC Canada; 10 Biogen Inc., Cambridge, MA USA; 11 Brain and Mind Centre, University of Sydney, Sydney, NSW Australia; 12 Institute of Neuroscience and Medicine (INM-1), Research Centre Jülich, Jülich, Germany; 13 Department of Physiology and Pharmacology, University of Western Ontario, London, ON Canada; 14 Department of Neurology, Clinical Neuroscience Center, University Hospital Zurich, Zurich, Switzerland; 15 Department of Clinical and Experimental Epilepsy, UCL Queen Square Institute of Neurology, London, UK; 16 MRI Unit, Chalfont Centre for Epilepsy, Chalfont Saint Peter, UK; 17 Department of Medicine, Division of Anaesthesia, University of Cambridge, Addenbrooke’s Hospital, Cambridge, UK; 18 Department of Clinical Neurosciences, Wolfson Brain Imaging Centre, University of Cambridge, Addenbrooke’s Hospital, Cambridge, UK; 19 King’s College London and Guy’s and St. Thomas’ PET Centre, Division of Imaging Sciences and Biomedical Engineering, King’s College London, London, UK; 20 Department of Psychiatry, Royal’s Institute of Mental Health Research, University of Ottawa, Ottawa, ON Canada; 21 Department of Psychoeducation and Psychology, University of Quebec in Outaouais, Gatineau, QC Canada; 22 Department of Psychiatry, University of Turku and Turku University Hospital, Turku, Finland; 23 Department of Clinical Medicine, University of Copenhagen, Copenhagen, Denmark; 24 C. and O. Vogt Institute for Brain Research, Medical Faculty, University Hospital Düsseldorf, Heinrich-Heine University Düsseldorf, Düsseldorf, Germany

**Keywords:** Network models, Neurotransmitters

## Abstract

Neurotransmitter receptors support the propagation of signals in the human brain. How receptor systems are situated within macro-scale neuroanatomy and how they shape emergent function remain poorly understood, and there exists no comprehensive atlas of receptors. Here we collate positron emission tomography data from more than 1,200 healthy individuals to construct a whole-brain three-dimensional normative atlas of 19 receptors and transporters across nine different neurotransmitter systems. We found that receptor profiles align with structural connectivity and mediate function, including neurophysiological oscillatory dynamics and resting-state hemodynamic functional connectivity. Using the Neurosynth cognitive atlas, we uncovered a topographic gradient of overlapping receptor distributions that separates extrinsic and intrinsic psychological processes. Finally, we found both expected and novel associations between receptor distributions and cortical abnormality patterns across 13 disorders. We replicated all findings in an independently collected autoradiography dataset. This work demonstrates how chemoarchitecture shapes brain structure and function, providing a new direction for studying multi-scale brain organization.

## Abstract (web summary)

Hansen et al. compile and share an atlas of neurotransmitter receptor/transporter densities in the human cortex and show that receptor achitecture reflects brain structure, function, dynamics, cognitive specialization and disease vulnerability.

## Main

Neurotransmitter receptors are heterogeneously distributed across the neocortex and respond to the binding of a neurotransmitter. By modulating the excitability and firing rate of the cell, neurotransmitter receptors effectively mediate the transfer and propagation of electrical impulses. As such, neurotransmitter receptors drive synaptic plasticity, modify neural states and ultimately shape network-wide communication^1^. These receptors are diverse in their structure and function: receptors may be ionotropic or metabotropic, may be composed of multiple subunits, may exert facilitatory or inhibitory influence on the circuit and are coupled to different downstream biochemical pathways.

How spatial distributions of different neurotransmitter receptors relate to brain structure and shape brain function at the system level remains unknown. Recent technological advances allow for high-resolution reconstructions of the brain’s wiring patterns. These wiring patterns display non-trivial architectural features, including specialized network modules that support the segregation of information^2^ as well as densely interconnected hub regions that support the integration of information^3^. The spatial arrangement of neurotransmitter receptors on this network presumably guides the flow of information and the emergence of cognitive function. Therefore, understanding the link between structure and function is inherently incomplete without a comprehensive map of the chemoarchitecture of the brain^4,5^.

A primary obstacle to studying the relative density distributions of receptors across multiple neurotransmitter systems is the lack of comprehensive openly accessible datasets. An important exception is the autoradiography dataset of 15 neurotransmitter receptors and receptor-binding sites, collected in three postmortem brains^4,6^. However, these autoradiographs are available in only 44 cytoarchitectonically defined cortical areas. Alternatively, positron emission tomography (PET) can estimate in vivo receptor concentrations across the whole brain. Despite the relative ease of mapping receptor densities using PET, there are, nonetheless, difficulties in constructing a comprehensive PET dataset of neurotransmitter receptors. Due to the radioactivity of the injected PET tracer, mapping multiple different receptors in the same individual is not considered a safe practice. Combined with the fact that PET image acquisition is relatively expensive, cohorts of control subjects are small and typically include only one or two tracers. Therefore, constructing a comprehensive atlas of neurotransmitter receptor densities across the brain requires extensive data-sharing efforts from multiple research groups^7–11^.

Here we curate and share an atlas of PET-derived whole-brain neurotransmitter receptor maps from 19 unique neurotransmitter receptors, receptor-binding sites and transporters, across nine different neurotransmitter systems and more than 1,200 healthy individuals, available at https://github.com/netneurolab/hansen_receptors. We use multiple imaging modalities to comprehensively situate cortical neurotransmitter receptor densities within micro-scale and macro-scale neural architectures. Using diffusion-weighted magnetic resonance imaging (MRI) and functional MRI, we show that neurotransmitter receptor densities follow the organizational principles of the brain’s structural and functional connectomes. Moreover, we found that neurotransmitter receptor densities shape magnetoencephalography (MEG)-derived oscillatory neural dynamics. To determine how neurotransmitter receptor distributions affect cognition and disease, we mapped receptor densities to meta-analytic (Neurosynth-derived) functional activations, where we uncovered a spatially co-varying axis of neuromodulators and mood-related processes. Next, we linked receptor distributions to ENIGMA-derived patterns of cortical atrophy across 13 neurological, psychiatric and neurodevelopmental disorders, uncovering specific receptor–disorder links. We validated our findings and extended the scope of the investigation to additional receptors using an independently collected autoradiography neurotransmitter receptor dataset^6^. Altogether, we demonstrate that, across spatial and temporal scales, chemoarchitecture consistently plays a key role in brain function.

## Results

**Fig. 1:**
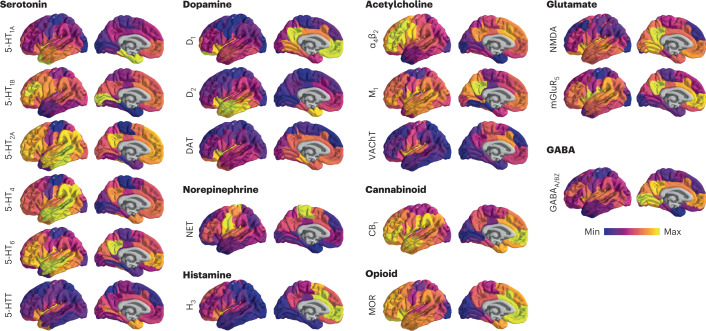
PET images of neurotransmitter receptors and transporters. PET tracer images were collated and averaged to produce mean receptor distribution maps of 19 different neurotransmitter receptors and transporters across nine different neurotransmitter systems and a combined total of more than 1,200 healthy participants.

A comprehensive cortical profile of neurotransmitter receptor densities was constructed by collating PET images from a total of 19 different neurotransmitter receptors, transporters and receptor-binding sites across nine different neurotransmitter systems, including dopamine, norepinephrine, serotonin, acetylcholine, glutamate, GABA, histamine, cannabinoid and opioid (Fig. 1). All PET images were acquired in healthy participants (see Table 1 for a complete list of receptors and transporters, corresponding PET tracers, ages and number of participants). A group-average tracer map was constructed across participants within each study. To mitigate variation in image acquisition and pre-processing, and to ease biological interpretability, all PET tracer maps were parcellated into the same 100 cortical regions and z-scored^12^. Note that, although the data include both cortical and subcortical data, we restricted our analyses to the cortex. In total, we present tracer maps for 19 unique neurotransmitter receptors and transporters from a combined total of 1,238 healthy participants, resulting in a 100 × 19 matrix of relative neurotransmitter receptor/transporter densities. Finally, we repeated all analyses in an independently collected autoradiography dataset of 15 neurotransmitter receptors (Supplementary Table 1 (ref. ^6^)) and across alternative brain parcellations^12^.

**Table 1:** Neurotransmitter receptors and transporters included in analyses. BP_ND_, non-displaceable binding potential; V_T_, tracer distribution volume; B_max_, density (pmol ml^−1^) converted from binding potential (5-HT) or distributional volume (GABA) using autoradiography-derived densities; SUVR, standard uptake value ratio. Values in parentheses (under *n*) indicate the number of females. Neurotransmitter receptor maps without citations refer to previously unpublished data. In those cases, contact information for the study principal investigator (PI) is provided in Supplementary Table 3. Supplementary Table 3 also includes more extensive methodological details, such as PET camera, number of males and females, modeling method, reference region, scan length and modeling notes. Asterisks indicate transporters

Receptor/transporter	Neurotransmitter	Tracer	Measure	*n*	Age	References
D_1_	Dopamine	[^11^C]SCH23390	BP_ND_	13 (7)	33 ± 13	Kaller et al.^58^
D_2_	Dopamine	[^11^C]FLB-457	BP_ND_	37 (20)	48.4 ± 16.9	Smith et al.^59,60^
D_2_	Dopamine	[^11^C]FLB-457	BP_ND_	55 (29)	32.5 ± 9.7	Sandiego et al.^59–63^
DAT^*^	Dopamine	[^123^I]-FP-CIT	SUVR	174 (65)	61 ± 11	Dukart et al.^64^
NET^*^	Norepinephrine	[^11^C]MRB	BP_ND_	77 (27)	33.4 ± 9.2	Ding et al.^65–68^
5-HT_1A_	Serotonin	[^11^C]WAY-100635	BP_ND_	35 (17)	26.3 ± 5.2	Savli et al.^69^
5-HT_1B_	Serotonin	[^11^C]P943	BP_ND_	65 (16)	33.7 ± 9.7	Gallezot et al.^70–76^
5-HT_1B_	Serotonin	[^11^C]P943	BP_ND_	23 (8)	28.7 ± 7.0	Savli et al.^69^
5-HT_2A_	Serotonin	[^11^C]Cimbi-36	B${}_{\max}$	29 (14)	22.6 ± 2.7	Beliveau et al.^9^
5-HT_4_	Serotonin	[^11^C]SB207145	B${}_{\max}$	59 (18)	25.9 ± 5.3	Beliveau et al.^9^
5-HT_6_	Serotonin	[^11^C]GSK215083	BP_ND_	30 (0)	36.6 ± 9.0	Radhakrishnan et al.^77,78^
5-HTT^*^	Serotonin	[^11^C]DASB	B${}_{\max}$	100 (71)	25.1 ± 5.8	Beliveau et al.^9^
α_4_β_2_	Acetylcholine	[^18^F]Flubatine	V_T_	30 (10)	33.5 ± 10.7	Hillmer et al.^79,80^
M_1_	Acetylcholine	[^11^C]LSN3172176	BP_ND_	24 (11)	40.5 ± 11.7	Naganawa et al.^81^
VAChT^*^	Acetylcholine	[^18^F]FEOBV	SUVR	4 (1)	37 ± 10.2	PI: Tuominen, L. & Guimond, S.
VAChT^*^	Acetylcholine	[^18^F]FEOBV	SUVR	18 (13)	66.8 ± 6.8	Aghourian et al.^82^
VAChT^*^	Acetylcholine	[^18^F]FEOBV	SUVR	5 (1)	68.3 ± 3.1	Bedard et al.^83^
VAChT^*^	Acetylcholine	[^18^F]FEOBV	SUVR	3 (3)	66.6 ± 0.94	PI: Schmitz, T. W. & Spreng, R. N.
NMDA	Glutamate	[^18^F]GE-179	V_T_	29 (8)	40.9 ± 12.7	Galovic et al.^84–86^
mGluR_5_	Glutamate	[^11^C]ABP688	BP_ND_	73 (48)	19.9 ± 3.04	Smart et al.^52^
mGluR_5_	Glutamate	[^11^C]ABP688	BP_ND_	22 (10)	67.9 ± 9.6	PI: Rosa-Neto, P. & Kobayashi, E.
mGluR_5_	Glutamate	[^11^C]ABP688	BP_ND_	28 (13)	33.1 ± 11.2	DuBois et al.^87^
GABA_A/BZ_	GABA	[^11^C]Flumazenil	B${}_{\max}$	16 (9)	26.6 ± 8	Nørgaard et al.^8^
H_3_	Histamine	[^11^C]GSK189254	V_T_	8 (1)	31.7 ± 9.0	Gallezot et al.^88^
CB_1_	Cannabinoid	[^11^C]OMAR	V_T_	77 (28)	30.0 ± 8.9	Normandin et al.^89–92^
MOR	Opioid	[^11^C]Carfentanil	BP_ND_	204 (72)	32.3 ± 10.8	Kantonen et al.^93^

### Receptor distributions reflect structural and functional organization

**Fig. 2:**
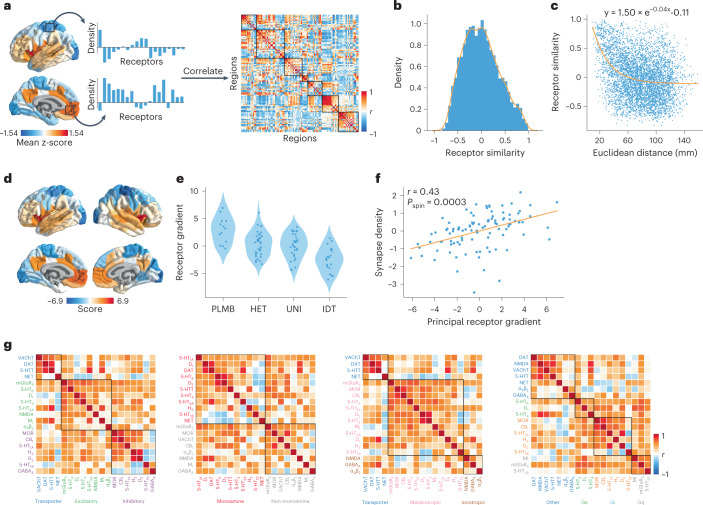
Constructing a cortical neurotransmitter receptor and transporter atlas. PET maps for 19 different neurotransmitter receptors and transporters were z-scored and collated into a single neurotransmitter receptor atlas. **a**, For each pair of brain regions, the receptor density profiles are correlated (Pearsonʼs *r*) to construct the receptor similarity matrix (ordered according to the Yeo–Krienen intrinsic networks: frontoparietal, default mode, dorsal attention, limbic, ventral attention, somatomotor and visual^23^). **b**, Receptor similarity is approximately normally distributed. **c**, Receptor similarity decays exponentially with the Euclidean distance between centroid coordinates of brain regions. **d**, The first principal component of receptor density. **e**, The first principal gradient of receptor density stratified by classes of laminar differentiation reveals a gradient from idiotypic regions to paralimbic regions (one-way ANOVA *F* = 15.82, *P* = 1.95 × 10^−8^; PLMB, paralimbic; HET, heteromodal; UNI, unimodal; IDT, idiotypic)^17^. **f**, The principal receptor gradient is significantly correlated with synapse density (measured using the synaptic vesicle glycoprotein 2A-binding [^11^C]-UCBJ PET tracer; Pearsonʼs *r*(98) = 0.44, *P*_spin_ = 0.0003, CI = [0.26, 0.58], two-tailed). **g**, Pearsonʼs correlations between pairs of receptor/transporter distributions are shown stratified by excitatory versus inhibitory, monoamine versus non-monoamine, ionotropic versus metabotropic and Gs-coupled versus Gi-coupled versus Gq-coupled metabotropic receptors.

To quantify the potential for two brain regions to be similarly modulated by endogenous or exogenous input, we computed the correlation of receptor/transporter fingerprints between pairs of brain regions (Fig. 2a). Hereafter, we refer to this quantity as ‘receptor similarity’, analogous to other commonly used measures of inter-regional attribute similarity, including anatomical covariance^13^, morphometric similarity^14^, gene coexpression^15^, temporal profile similarity^16^ and microstructural similarity^17^. Receptor similarity is approximately normally distributed (Fig. 2b) and decreases exponentially with Euclidean distance, supporting the notion that proximal neural elements share similar microarchitecture (Fig. 2c; refs. ^18,19^). We confirm that no single receptor or transporter exerts undue influence on the receptor similarity matrix (see the ‘Sensitivity and robustness analyses’ section).

Receptor similarity addresses the between-region similarity of receptor fingerprints. To complement this, we calculated the first principal component of receptor density, which represents a regional quantification of receptor similarity (Fig. 2d). This gradient separates insular and cingulate cortex from somatomotor and posterior parietal regions and resembles the macaque principal receptor expression gradient^20^. The first principal component differentiates laminar classes, supporting the notion that receptor expression strongly depends on lamination (Fig. 2e; one-way ANOVA *F* = 15.82, *P* = 1.95 × 10^−8^; ref. ^21^). Additionally, we found a significant correlation between the receptor gradient and synapse density, consistent with the finding that the macaque receptor gradient increases with the number of dendritic spines (Fig. 2f; Pearson’s *r*(98) = 0.44, *P*_spin_ = 0.0003, confidence interval (CI) = [0.26, 0.58], two-tailed)^20^. For completeness, we stratified receptors by biological mechanisms (excitatory/inhibitory, ionotropic/metabotropic and Gs-/Gi-/Gq-coupled metabotropic pathways) and neurotransmitter protein structure (monoamine/non-monoamine) to provide additional insight about the underlying biological pathways (Fig. 2g).

**Fig. 3:**
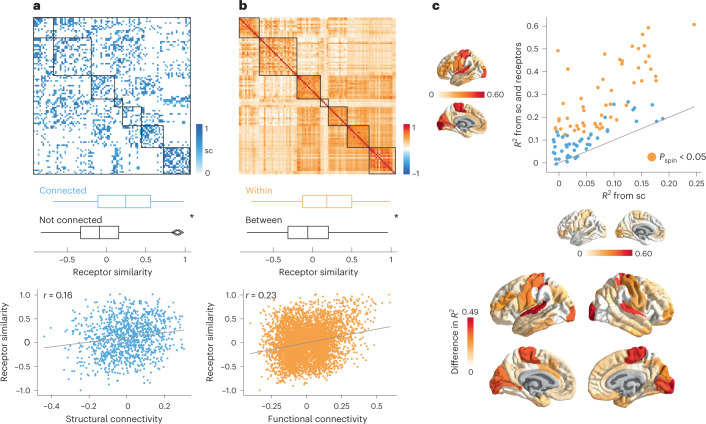
Receptor distributions reflect structural and functional organization. **a**, Top: group consensus weighted structural connectivity matrix. Middle: Receptor similarity is significantly greater between regions that are physically connected, against distance- and edge length-preserving null structural connectivity matrices (*P* = 0.0001, two-tailed *N*_connected_ = 1,136 edges, *N*_notconnected_ = 3,814 edges^22^). Bottom: Receptor similarity is significantly positively correlated with structural connectivity, after distance regression (Pearsonʼs *r*(1134) = 0.16, *P* = 1.6 × 10^−8^, CI = [0.11, 0.23], two-sided). **b**, Top: group-average functional connectivity matrix. Middle: Receptor similarity is significantly greater within regions in the same functional network (*P*_spin_ = 0.001, two-tailed, *N*_within_ = 762 edges, *N*_between_ = 4,188 edges). Bottom: Receptor similarity is positively correlated with functional connectivity (Pearsonʼs *r*(4948) = 0.23, *P* = 7.1 × 10^−61^, CI = [0.20, 0.26], two-sided). **c**, Regional structure–function coupling was computed as the fit (${R}_{{{{\rm{adj}}}}}^{2}$) between communicability of the weighted structural connectome and functional connectivity. Top: Structure–function coupling at each brain region is plotted when receptor similarity is excluded (*x*-axis) and included (*y*-axis) in the model. Yellow points indicate brain regions where receptor information significantly augments structure–function coupling (*P*_spin_ < 0.05, FDR-corrected, one-sided). Bottom: the difference in adjusted *R*^2^ when receptor similarity is and is not included in the regression model. Asterisks in **a** and **b** denote significance. Box plots in **a** and **b** represent the 1st, 2nd (median) and 3rd quartiles; whiskers represent the non-outlier endpoints of the distribution; and diamonds represent outliers. Connectomes in **a** and **b** are ordered according to the Yeo–Krienen intrinsic networks (order: frontoparietal, default mode, dorsal attention, limbic, ventral attention, somatomotor and visual)^23^. sc, structural connectivity.

Using group-consensus structural and resting-state functional connectomes from the Human Connectome Project (HCP), we show that neurotransmitter receptor organization reflects structural and functional connectivity. Specifically, we found that receptor similarity is greater between pairs of brain regions that are structurally connected, suggesting that anatomically connected areas are likely to be co-modulated (Fig. 3a). To ensure that the observed relationship between structural connections and receptor similarity is not due to spatial proximity or network topography, we assessed significance against density-, degree- and edge length-preserving surrogate structural connectivity matrices (*P* = 0.0001, 10,000 repetitions^22^). Additionally, we found that receptor similarity is significantly correlated with structural connectivity, after regressing Euclidean distance from both modalities (Pearson’s *r*(1134) = 0.16, *P* = 1.6 × 10^−8^, CI = [0.11, 0.23], two-sided).

Likewise, receptor similarity is significantly greater between brain regions that are within the same intrinsic networks than between different intrinsic networks, according to the Yeo–Krienen seven-network classification (*P*_spin_ = 0.001, 10,000 repetitions; Fig. 3b (ref. ^23^)). This suggests that areas that are in the same cognitive system tend to have similar receptor profiles^4^. Significance was assessed non-parametrically by permuting the intrinsic network affiliations while preserving spatial autocorrelation (‘spin test’; refs. ^24,25^). We also found that receptor similarity is significantly correlated with functional connectivity, after regressing Euclidean distance from both matrices (Pearsonʼs *r*(4948) = 0.23, *P* = 7.1 × 10^−61^, CI = [0.20, 0.26], two-sided). In other words, we observed that brain regions with similar receptor and transporter composition show greater functional co-activation. Collectively, these results demonstrate that receptor profiles are systematically aligned with patterns of structural and functional connectivity above and beyond spatial proximity, consistent with the notion that receptor profiles guide inter-regional signaling.

Because neurotransmitter receptor and transporter distributions are organized according to structural and functional architectures, we next asked whether receptor/transporter distributions might augment the coupling between brain structure and function. To quantify structure–function coupling, we relied on the communicability of the weighted structural connectome (see results using alternative methods in Supplementary Fig. 1). Communicability represents a form of decentralized diffusive communication on the structural connectome^26^ and has been previously shown to mediate the link between brain structure and function^27^. Structure–function coupling at every brain region is defined as the adjusted *R*^2^ of a simple linear regression model that fits regional communicability to regional functional connectivity. We then included regional receptor similarity as an independent variable, to assess how receptor information changes structure–function coupling. Significance was assessed against a null distribution of adjusted *R*^2^ from a model that adds a rotated regional receptor similarity vector (10,000 repetitions, one-sided, false disovery rate (FDR)-corrected). Next, we cross-validated each regression model using a distance-dependent method that was previously developed in-house (Supplementary Fig. 2; see Methods for details^28^). We found that including receptor profiles as an input variable alongside brain structure significantly improves the prediction of regional functional connectivity in unimodal areas and the paracentral lobule (Fig. 3c).

### Receptor profiles shape oscillatory neural dynamics

**Fig. 4:**
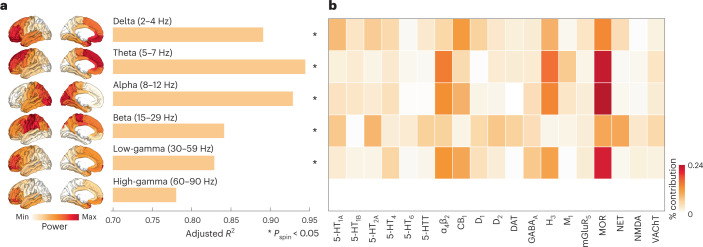
Receptor profiles shape oscillatory neural dynamics. We fit a multi-linear regression model that predicts MEG-derived power distributions from receptor distributions. **a**, Receptor distributions closely correspond to all six MEG-derived power bands ($0.78\le {R}_{{{{\rm{adj}}}}}^{2}(80)\le 0.94$). The significance of each model is assessed against a spatial permutation-preserving null model and corrected for multiple comparisons (FDR correction). Asterisks denote significant models (FDR-corrected *P*_spin_ < 0.05, one-tailed). Delta ${R}_{{{{\rm{adj}}}}}^{2}(80)=0.89$, *P*_spin_ = 0.03; theta ${R}_{{{{\rm{adj}}}}}^{2}(80)=0.94$, *P*_spin_ = 0.0006; alpha ${R}_{{{{\rm{adj}}}}}^{2}(80)=0.93$, *P*_spin_ = 0.0006; beta ${R}_{{{{\rm{adj}}}}}^{2}(80)=0.84$, *P*_spin_ = 0.008; low-gamma ${R}_{{{{\rm{adj}}}}}^{2}(80)=0.83$, *P*_spin_ = 0.04; and high-gamma ${R}_{{{{\rm{adj}}}}}^{2}(80)=0.78$, *P*_spin_ = 0.16. **b**, Dominance analysis distributes the fit of the model across input variables such that the contribution of each variable can be assessed and compared to other input variables. The percent contribution of each input variable is defined as the variableʼs dominance normalized by the total fit (${R}_{{{{\rm{adj}}}}}^{2}$) of the model. Note that dominance analysis is not applied to the input variables of non-significant models (that is, high-gamma).

Given that neurotransmitter receptors modulate the firing rates of neurons and, therefore, population activity, we sought to relate the cortical patterning of neurotransmitter receptors to neural oscillations. We used MEG power spectra across six canonical frequency bands from the HCP^29,30^. We fit a multiple linear regression model that predicts the cortical power distribution of each frequency band from neurotransmitter receptor and transporter densities. We then cross-validated the model using a distance-dependent method (Supplementary Fig. 3). In addition to the cross-validation, we assessed the significance of each model against a spin-permuted null model (10,000 repetitions) and found that all models except high-gamma are significant after FDR correction (*P*_spin_ < 0.05, one-sided). We found a close fit between receptor densities and MEG-derived power ($0.78\le {R}_{{{{\rm{adj}}}}}^{2}\le 0.94$; Fig. 4a), suggesting that overlapping spatial topographies of multiple neurotransmitter systems may ultimately manifest as coherent oscillatory patterns.

To identify independent variables (receptors/transporters) that contribute most to the fit, we applied dominance analysis, a technique that assigns a proportion of the final ${R}_{{{{\rm{adj}}}}}^{2}$ to each independent variable to the statistically significant models^31^. Dominance was normalized by the total fit of the model (${R}_{{{{\rm{adj}}}}}^{2}$), such that dominance is comparable across models (Fig. 4b). We found that, compared to other receptors, the spatial distribution of MOR (opioid), H_3_ (histamine) and α_4_β_2_ make a large contribution to the fit between receptors and lower-frequency (theta and alpha) as well as low-gamma power bands^32,33^. Interestingly, we found a prominence of ionotropic receptors when we replicated the analysis in the autoradiography dataset (see the ‘Replication using autoradiography’ section and Supplementary Fig. 4). Additionally, when we stratified dominance by receptor classes, we found that inhibitory, non-monoamine and Gi-coupled receptors are more dominant than excitatory, monoamine and Gs-/Gq-coupled receptors, respectively (Supplementary Fig. 5a).

### Mapping receptors to cognitive function

Previously, we showed that receptor and transporter distributions follow the structural and functional organization of the brain and that receptors are closely linked to neural dynamics. In this and the next subsections, we investigate how the spatial distribution of neurotransmitter receptors and transporters correspond to cognitive processes and disease vulnerability.

We used Neurosynth to derive 123 meta-analytic task activation maps, which represent the probability that specific brain regions are activated during multiple cognitive tasks^34^. We applied partial least squares (PLS) analysis to identify a multivariate mapping between neurotransmitter receptors/transporters and functional activation maps.

**Fig. 5:**
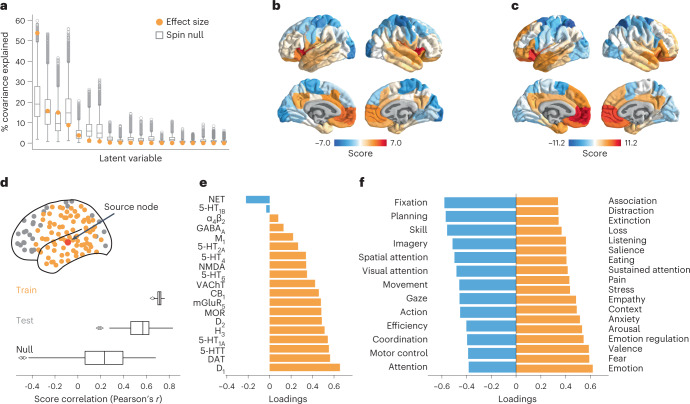
Mapping receptors to cognitive function. **a**, Using PLS analysis, we found a significant latent variable that accounts for 54% of the covariation between receptor distributions and Neurosynth-derived cognitive functional activation (*P*_spin_ = 0.010, 10,000 repetitions, one-sided). **b**,**c**, This latent variable represents a pattern of co-activation between receptors (‘receptor scores’) and cognitive terms (‘cognitive scores’). **d**, The PLS model was cross-validated using a method that stratifies the training set (yellow points) and test set (gray points) based on the distance between each node to a source node (red point), and the procedure is repeated such that each brain region is assigned as the source node once (100 repetitions). The significance of the mean out-of-sample test set correlation was assessed against a null distribution of mean correlation constructed by rotating the receptor density matrix before the PLS analysis (see Methods for details). **e**, Receptor loadings are computed as the correlation (Pearsonʼs *r*) between each receptorʼs distribution across the cortex and the PLS-derived scores and can be interpreted as the contribution of each receptor to the latent variable. **f**, Similarly, cognitive loadings are computed as the correlation (Pearsonʼs *r*) between each termʼs functional activation across brain regions and the PLS-derived scores and can be interpreted as the cognitive processes that contribute most to the latent variable. Here, only the 25% most positively and negatively loaded cognitive processes are shown. For all stable cognitive loadings, see Supplementary Fig. 6, and, for all 123 cognitive processes included in the analysis, see Supplementary Table 2. Bounds of the box plots in **a** and **d** represent the 1st (25%) and 3rd (75%) quartiles; the center line represents the median; whiskers represent the non-outlier minima and maxima of the distribution; and open circles represent outliers.

PLS analysis extracted a significant latent variable relating receptor/transporter densities to functional activation across the brain (*P*_spin_ = 0.010, one-tailed). The latent variable represents the dominant spatial pattern of receptor distributions (receptor weights) and functional activations (cognitive weights) that together capture 54% of the covariance between the two datasets (Fig. 5a). Projecting the receptor density (functional activation) matrix back onto the receptor (cognitive) weights reflects how well a brain area exhibits the receptor and cognitive weighted pattern, which we refer to as ‘receptor scores’ and ‘cognitive scores’, respectively (Fig. 5b,c). The receptor and cognitive score patterns reveal a sensory-fugal spatial gradient, separating limbic, paralimbic and insular cortices from visual and somatosensory cortices. We then cross-validated the correlation between receptor and cognitive scores using a distance-dependent method (Fig. 5d, mean out-of-sample Pearsonʼs *r*(98) = 0.54, *P*_spin_ = 0.046, one-sided). This result demonstrates a link between receptor distributions and cognitive specialization that is perhaps mediated by laminar differentiation and synaptic hierarchies.

To identify the receptors and cognitive processes that contribute most to the spatial pattern in Fig. 5b,c, we correlated each variable with the score pattern (Fig. 5e–f; for all stable term loadings, see Supplementary Fig. 6). This results in a ‘loading’ for each receptor and cognitive process, where positively loaded receptors co-vary with positively loaded cognitive processes in positively scored brain regions and vice versa for negative loadings. Interestingly, we found that almost all receptors/transporters have positive loading, with metabotropic dopaminergic and serotonergic receptors having the greatest loadings (Fig. 5e and Supplementary Fig. 5b). The cognitive processes with large positive loadings are enriched for emotional and affective processes such as ‘emotion’, ‘fear’ and ‘valence’. This suggests that the combination of serotonergic and dopaminergic receptor distributions co-vary with mood-related functional activation in insular and limbic regions, consistent with the role of serotonin and dopamine neurotransmitter systems in mood processing and mood disorders^35^. On the other hand, we found that only NET has stable negative loading and that it spatially co-varies with functions such as ‘fixation’, ‘planning’ and ‘skill’ in primarily unimodal regions. This is consistent with the notion that norepinephrine systems are involved in integrative functions that require coordination across segregated brain regions^1^. Collectively, these results demonstrate a direct link between cortex-wide molecular receptor distributions and functional specialization.

### Mapping receptors and transporters to disease vulnerability

**Fig. 6:**
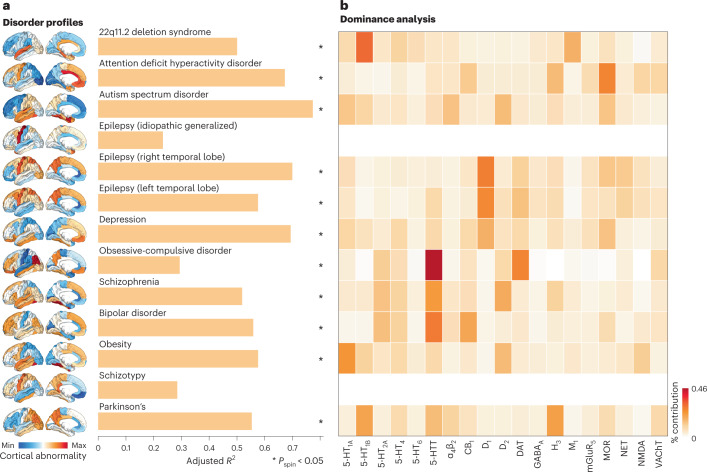
Mapping receptors to disease vulnerability. Using a multi-linear model, neurotransmitter receptor/transporter distributions were fit to patterns of cortical abnormality for 13 neurological, psychiatric and neurodevelopmental disorders, collected by the ENIGMA consortium. **a**, The significance of each model is assessed using a spatial autocorrelation-preserving null model and is corrected for multiple comparisons (FDR). Asterisks denote significant models (FDR-corrected *P*_spin_ < 0.05, one-sided). 22q11.2 deletion ${R}_{{{{\rm{adj}}}}}^{2}(48)=0.50$, *P*_spin_ = 0.02; ADHD ${R}_{{{{\rm{adj}}}}}^{2}(48)=0.67$, *P*_spin_ = 0.02; autism ${R}_{{{{\rm{adj}}}}}^{2}(48)=0.77$, *P*_spin_ = 0.02; epilepsy (IGE) ${R}_{{{{\rm{adj}}}}}^{2}(48)=0.23$, *P*_spin_ = 0.17; epilepsy (right) ${R}_{{{{\rm{adj}}}}}^{2}(48)=0.70$, *P*_spin_ = 0.02; epilepsy (left) ${R}_{{{{\rm{adj}}}}}^{2}(48)=0.58$, *P*_spin_ = 0.02; depression ${R}_{{{{\rm{adj}}}}}^{2}(48)=0.69$, *P*_spin_ = 0.01; OCD ${R}_{{{{\rm{adj}}}}}^{2}(48)=0.29$, *P*_spin_ = 0.02; schizophrenia ${R}_{{{{\rm{adj}}}}}^{2}(48)=0.52$, *P*_spin_ = 0.02; BD ${R}_{{{{\rm{adj}}}}}^{2}(48)=0.56$, *P*_spin_ = 0.01; obesity ${R}_{{{{\rm{adj}}}}}^{2}(48)=0.58$, *P*_spin_ = 0.02; schizotypy ${R}_{{{{\rm{adj}}}}}^{2}(48)=0.29$, *P*_spin_ = 0.32; and PD ${R}_{{{{\rm{adj}}}}}^{2}(48)=0.55$, *P*_spin_ = 0.02. **b**, Dominance analysis distributes the fit of the model across input variables such that the contribution of each variable can be assessed and compared to other input variables. The percent contribution of each input variable is defined as the variableʼs dominance normalized by the total fit (R${}_{{{{\rm{adj}}}}}^{2}$) of the model. Note that dominance analysis is not applied to the input variables of non-significant models (that is, IGE and schizotypy) and that this analysis is conducted using the Desikan–Killiany atlas because this is the only representation of ENIGMA datasets.

Neurotransmitter receptors and transporters are implicated in multiple diseases and disorders. Identifying the neurotransmitter receptors/transporters that correspond to specific disorders is important for developing new therapeutic drugs. We, therefore, sought to relate neurotransmitter receptors and transporters to patterns of cortical abnormality across a range of neurological, developmental and psychiatric disorders. We used datasets from the ENIGMA consortium for a total of 13 disorders, including 22q11.2 deletion syndrome, attention deficit hyperactivity disorder (ADHD), autism spectrum disorder (ASD), idiopathic generalized epilepsy (IGE), right and left temporal lobe epilepsy, depression, obsessive-compulsive disorder (OCD), schizophrenia, bipolar disorder (BD), obesity, schizotypy and Parkinson’s disease (PD). We then fit a multiple regression model that predicts each disorder’s cortical abnormality pattern from receptor and transporter distributions (Fig. 6). We assessed the significance of each model fit against an FDR-corrected one-sided spatial autocorrelation-preserving null model and evaluated each model using distance-dependent cross-validation (Supplementary Fig. 7). Figure 6a shows how receptor distributions map onto cortical abnormaltiy patterns across multiple disorders. We found that some disorders are more heavily influenced by receptor distribution than others ($0.23 < {R}_{{{{\rm{adj}}}}}^{2} < 0.77$). IGE and schizotypy show low and non-significant correspondence with receptor distributions, whereas ADHD, autism and temporal lobe epilepsies show greater correspondence with receptor distributions. The dominance analysis in Fig. 6b shows the contribution of each input variable to the fit of the model, normalized by the total fit (adjusted *R*^2^). Interestingly, we found that serotonin transporter (5-HTT) distributions contribute more to OCD, schizophrenia and BD profiles than any other receptors. Furthermore, the mu-opioid receptor is the strongest contributor of ADHD cortical abnormalities, consistent with findings from animal models^36^. We also note that, in some cases, the analyses do not necessarily recover the expected relationships. For instance, in PD, the dopamine receptors are not implicated, likely because the analysis was restricted to cortex only. Additionally, serotonin receptors do not make large contributions to depression, possibly because changes in cortical thickness do not directly measure the primary pathophysiology associated with some brain diseases. Although this analysis points to mappings between receptors and disorder profiles, we found no significant differential contribution of receptor classes to disorder profiles (Supplementary Fig. 5c). Our results present an initial step toward a comprehensive ‘look-up table’ that relates neurotransmitter systems to multiple brain disorders.

### Replication using autoradiography

In the present report, we comprehensively situate neurotransmitter receptor and transporter densities within the brain’s structural and functional architecture. However, estimates for neurotransmitter receptor densities are acquired from PET imaging alone, and the way in which densities are quantified varies across radioligands, image acquisition protocols and pre-processing. Autoradiography is an alternative technique to measure receptor density and captures local densities at a defined number of postmortem brain sections. Due to the high cost and labor intensity of acquiring autoradiographs, there does not yet exist a complete autoradiography three-dimensional (3D) cross-cortex atlas of receptors.

Nonetheless, we repeated the analyses in an autoradiography dataset of 15 neurotransmitter receptors across 44 cytoarchitectonically defined cortical areas, from three postmortem brains^6,37^. This set of 15 neurotransmitter receptors consists of a diverse set of ionotropic and metabotropic receptors, including excitatory glutamate, acetylcholine and norepinephrine receptors (see Supplementary Table 1 for a complete list of receptors). Notably, eight of the 15 receptors in the autoradiography dataset are not included in the PET dataset, which precludes direct comparisons between the two datasets. Receptor similarity is shown in Supplementary Fig. 8a. Despite the alternate set of neurotransmitter receptors, we found that autoradiography-derived receptor similarity is significantly correlated with PET-derived receptor similarity (Pearson’s *r*(1033) = 0.38, *P* = 6.7 × 10^−38^, CI = [0.33, 0.44]; Supplementary Fig. 8a) and decays exponentially with Euclidean distance. Additionally, autoradiography-derived and PET-derived receptor gradients are correlated (Pearson’s *r*(44) = 0.51, *P*_perm_ = 0.0001, CI = [0.26, 0.70], two-sided). Next, we found that autoradiography-derived receptor densities follow similar architectural patterns as the PET-derived receptor densities. Receptor similarity is non-significantly greater between structurally connected brain regions (*P* = 0.19) and significantly correlated with structural connectivity (Pearson’s *r*(329) = 0.39, *P* = 1.4 × 10^−13^, CI = [0.30, 0.48]; Supplementary Fig. 8d). It is also significantly greater in regions within the same intrinsic network (*P*_spin_ = 0.03) and is significantly correlated with functional connectivity (Pearson’s *r*(1033) = 0.21, *P* = 1.1 × 10^−12^, CI = [0.16, 0.28]; Supplementary Fig. 8e). As before, receptor information augments structure–function coupling in visual, paracentral and somatomotor regions (Supplementary Fig. 8f). Finally, we show correlations of receptor density distribution between every pair of receptors in Supplementary Fig. 8g.

Because the autoradiography dataset has a more diverse set of ionotropic and metabotropic receptors, we also asked whether we would observe a prominence of ionotropic receptors for MEG oscillations. When we fit the 15 autoradiography neurotransmitter receptors to MEG power, we found that AMPA, NMDA, GABA_A_ and α_4_β_2_—all ionotropic receptors—are most dominant (Supplementary Fig. 4). This confirms that the fast oscillatory dynamics captured by MEG are closely related to the fluctuations in neural activity modulated by ionotropic neurotransmitter receptors.

Finally, we repeated analyses mapping receptor densities to cognitive functional activation and disease vulnerability. We found a similar topographic gradient linking autoradiography-derived receptor densities to Neurosynth-derived functional activations (Supplementary Fig. 9a). Indeed, PET-derived and autoradiography-derived receptor and cognitive scores are correlated (Supplementary Fig. 8b; Pearson’s *r* = −0.50, *P*_perm_ = 0.0002, CI = [−0.69, −0.26] for receptor scores; Pearson’s *r* = −0.75, *P*_perm_ = 0.0001, CI = [−0.86, −0.60] for cognitive scores). We also found consistencies regarding the loadings of receptors (Supplementary Fig. 9c) and cognitive processes (Supplementary Fig. 9d). Next, when we mapped autoradiography-derived receptor densities to cortical abnormality patterns of multiple disorders, we found prominent associations with receptors that were not included in the PET dataset, including a relationship between the ionotropic glutamate receptor kainate and depression (Supplementary Fig. 10).

### Sensitivity and robustness analyses

Finally, to ensure that results are not influenced by specific methodological choices, we repeated analyses using different parcellation resolutions and different receptor subsets, and we compared alternative PET tracers to the chosen PET tracers in the present report. Due to the low spatial resolution of PET tracer binding, we opted to present our main results using a coarse resolution of 100 cortical regions^12^. However, when using a parcellation resolution of 200 and 400 cortical regions, we found that the mean receptor density and receptor similarity remains consistent (Supplementary Fig. 11). We next asked whether any single receptor or transporter disproportionately influences receptor similarity. To test this, we iteratively removed a single receptor/transporter from the dataset and recomputed the receptor similarity matrix. These 19 different receptor similarity matrices are all highly correlated with the original similarity matrix (Pearson’s *r*(4948) > 0.98), confirming that the correspondence between regional receptor profiles is not driven by a single neurotransmitter receptor/transporter.

We also tested whether participant age affects the reported results. However, only mean age of individuals included in each tracer map was available. Therefore, we fit a linear model between the mean age of scanned participants contributing to each receptor/transporter tracer map and the z-scored receptor/transporter density, for each brain region separately. We then subtracted the relationship with age from the original receptor densities, resulting in an age-regressed receptor density matrix. We found that both age-regressed receptor density and age-regressed receptor similarity is highly correlated with the original receptor density/similarity (Pearson’s *r*(4948) = 0.78, *P* = 0, CI = [0.76, 0.79] and Pearson’s *r*(4948) = 0.984, *P* = 0, CI = [0.982, 0.985], respectively; Supplementary Fig. 12), suggesting that age has a negligible effect on the reported findings. However, we note that this analysis is not sensitive to individual subject variability and that certain neurotransmitter receptor systems show changes in receptor availability with age^38–40^.

## Discussion

In the present report, we curate a comprehensive 3D atlas of 19 neurotransmitter receptors and transporters. We demonstrate that chemoarchitecture is a key layer of the multi-scale organization of the brain. Neurotransmitter receptor profiles closely align with the structural connectivity of the brain and mediate its link with function, including neurophysiological oscillatory dynamics and resting-state hemodynamic functional connectivity. The overlapping topographic distributions of these receptors ultimately manifest as patterns of cognitive specialization and disease vulnerability.

A key question in neuroscience remains how the brainʼs structural architecture gives rise to its function^41^. The relationship between whole-brain structure and function has been viewed through the lens of ‘connectomics’, in which the brain’s structural or functional architectures are represented by regional nodes interconnected by structural and functional links. The key assumption of this model is that nodes are homogenous, effectively abstracting away important microarchitectural differences between regions. The present work is part of an emerging effort to annotate the connectome with molecular, cellular and laminar attributes. Indeed, recent work has incorporated microarray gene transcription^28^, cell types^42^, myelination^19^, laminar differentiation^43^ and intrinsic dynamics^44^ into structural and functional models of the brain.

Neurotransmitter receptors and transporters are an important molecular annotation for bridging brain structure to brain function. Despite this, a comprehensive cortical map of neurotransmitter receptors has remained elusive due to numerous methodological and data-sharing challenges (but see the ongoing PET-BIDS effort as well as the OpenNeuro PET initiative at https://openneuropet.github.io/ (refs. ^10,11^)). The present study is an ongoing Open Science grassroots effort to assemble harmonized high-resolution normative images of receptors and transporters that can be used to annotate connectomic models of the brain. This work builds on previous initiatives to map receptor densities using autoradiography, which has discovered prominent gradients of receptor expression in both human and macaque brains^6,20,37^. Notably, we found consistent results between autoradiography and PET datasets, which is encouraging because the PET dataset consists of a different group of receptors and transporters and has the added advantage of providing in vivo whole-brain data in large samples of healthy young participants.

We found a prominent link between receptor distribution and both brain structure and function, which supports the idea that the emergent functional architecture strongly depends on the underlying chemoarchitecture^4^. Interestingly, we found that the canonical electrophysiological frequency bands can be captured by the overlapping topographies of multiple receptors, consistent with the notion that receptors influence function by tuning gain and synchrony between neuronal populations^45^. Because receptors are correlated with multiple features of brain structure and function, a natural next question is how receptor distributions relate to psychological processes. We found a multivariate mapping between receptor profiles and cognitive activations. Interestingly, although individual receptors have been associated with specific functions (for example, D1 and selective attention^46^), our findings suggest that the combined spatial distribution of serotonergic and dopaminergic receptors underlie patterns of cognitive activation related to affect. Altogether, these results offer clues about how multiple neurotransmitter systems collectively influence cognitive functions and present novel hypotheses that future causal studies can test.

Finally, we discovered a robust spatial concordance between multiple receptor maps and cortical abnormality profiles across a wide range of brain disorders. A key step toward developing therapies for specific syndromes is to reliably map them onto underlying neural systems. This goal is challenging because psychiatric and neurological nosology is built around clinical features rather than neurobiological mechanisms. Our results complement some previously established associations between disorders and neurotransmitter systems and also reveal new associations. For instance, we found that the serotonin transporter is the strongest contributor to schizophrenia and BD, consistent with the fact that mood disorders are often accompanied with abnormal serotonin signaling^47,48^. On the other hand, we found associations that have some preliminary support in the literature but, to our knowledge, have not been conclusively established and adopted into clinical practice, including histamine H_3_ in PD^49^, MOR in ADHD^36^ and D_1_ and NET in temporal lobe epilepsy^50,51^. Mapping disease phenotypes to receptor profiles will help to identify novel targets for pharmacotherapy. This analysis is restricted to a single perspective of disease pathology (cortical thinning/thickening) and should be expanded in future work to encompass other forms of disease presentation as well as the effects of age and pathology on receptor/transporter density.

Collectively, the main results in the present report aim to go beyond traditional one-to-one (that is, univariate) associations between receptors and brain function, toward considering how multiple neurotransmitter systems work together. The present report builds on the theories generated by previous neurochemical and pharmacological causal studies, and it is encouraging to see consistent results at the level of the whole brain, across multiple neurotransmitter systems and using different imaging modalities. Furthermore, the comprehensive approach of this study showcases novel associations that may not have been considered before. This large-scale characterization of receptor systems should be validated in, and will hopefully inspire, future causal studies, driving the cycle of discovery. Altogether, our data and analyses provide a framework that allows us to test predictions from the wider literature and consolidate knowledge about neurotransmitter systems.

Some potential avenues for future complementary research are to study how receptor architecture changes in healthy aging, across the sexes, and how they map onto subcortical structures. Indeed, dopamine D1 and D2 receptor availability is commonly acknowledged to decrease with age in the subcortex^38^; serotonin transporter and receptor density have been reported to be significantly lower in older adults^39^; and GABA_A_ density is reported to be higher in older adults^40^. Likewise, previously published literature has reported greater whole-brain glutamate receptor densities in men^52^, greater kappa-opioid receptor density in men^53^ and greater mu-opioid receptor density in women^54^. Finally, multiple neurotransmitter projection systems originate in the subcortex^1^, and neurodegenerative disease progression has been linked with abnormal subcortical receptor expression^55^. Ultimately, future research is necessary to characterize multi-system receptor distributions across age and sex and within subcortical structures.

The present work should be considered alongside some important methodological considerations. First, main analyses were conducted using PET images, which detect tracer uptake at a low spatial resolution and without laminar specificity. Although results were replicated using an autoradiography dataset, and in a finer parcellation resolution, a comprehensive atlas of laminar-resolved receptor density measurements is necessary to fully understand how regional variations in receptor densities affect brain structure and function^21^. Second, PET tracer maps were acquired around the world, in different participants, on different scanners and using specific image acquisition and processing protocols recommended for each individual radioligand^56,57^. To mitigate this challenge, we normalized the spatial distributions and focused only on analyses related to the relative spatial topographies of receptors as opposed to the absolute values. Third, the linear models used in the present analyses assume independence between observations and linear relationships between receptors; we, therefore, employed spatial autocorrelation-preserving null models to account for the spatial dependencies between regions throughout the report. Fourth, analyses were restricted to the cortex, obscuring the contributions of subcortical neuromodulatory systems. Fifth, although we repeated our analyses in an autoradiography dataset, eight of the 15 receptors included in the autoradiography dataset are not included in the PET datasets, and, therefore, a direct comparison between datasets was not possible. Altogether, a 3D whole-brain comprehensive neurotransmitter receptor density dataset constructed using autoradiographs would be a valuable complement to the present work^6,21^.

In summary, we assembled a normative 3D atlas of neurotransmitter receptors in the human brain. We systematically mapped receptors to connectivity, dynamics, cognitive specialization and disease vulnerability. Our work uncovers a fundamental organizational feature of the brain and provides new direction for a multi-scale systems-level understanding of brain structure and function.

## Methods

All code and data used to perform the analyses can be found at https://github.com/netneurolab/hansen_receptors. Volumetric PET images are included in neuromaps (https://github.com/netneurolab/neuromaps) where they can be easily converted between template spaces^94^.

### PET data acquisition

Volumetric PET images were collected for 19 different neurotransmitter receptors and transporters across multiple studies. To protect patient confidentiality, individual participant maps were averaged within studies before being shared. Details of each study, the associated receptor/transporter, tracer, number of healthy participants, age and reference with full methodological details can be found in Table 1. A more extensive table can be found in the supplementary material (Supplementary Table 3), which additionally includes the PET camera, number of males and females, PET modeling method, reference region, scan length, modeling notes and additional references, if applicable. In all cases, only healthy participants were scanned (*n* = 1,238; 718 males and 520 females). Images were acquired using best practice imaging protocols recommended for each radioligand^56^. Altogether, the images are an estimate proportional to receptor densities, and we, therefore, refer to the measured value (that is, binding potential and tracer distribution volume) simply as density. Note that the NMDA receptor tracer ([^18^F]GE-179) binds to open (that is, active) NMDA receptors^86,95^. PET images were all registered to the MNI-ICBM 152 non-linear 2009 (version c, asymmetric) template and then parcellated to 100, 200 and 400 regions according to the Schaefer atlas^12^. Receptors and transporters with more than one mean image of the same tracer (that is, 5-HT_1B_, D_2_, mGluR_5_ and VAChT) were combined using a weighted average after confirming that the images are highly correlated to one another (Supplementary Fig. 13a). Finally, each tracer map corresponding to each receptor/transporter was z-scored across regions and concatenated into a final region by receptor matrix of relative densities.

In some cases, more than one tracer map was available for the same neurotransmitter receptor/transporter. We show the comparisons between tracers in Supplementary Fig. 13b for the following neurotransmitter receptors/transporters: 5-HT1_A_^9,69^, 5-HT1_B_^9,69,70^, 5-HT2_A_^9,69,96^, 5-HTT^9,69^, CB_1_ (refs. (^89,97^)), D_2_ (refs. (^59,60,98,99^)), DAT^64,100^, GABA_A_^8,64^, MOR^93,101^ and NET^65,102^. Here, we make some specific notes: (1) 5-HTT and GABA_A_ involve comparisons between the same tracers (DASB and flumazenil, respectively), but one map is converted to density using autoradiography data (see ref. ^9^ and ref. ^8^) and the other is not^7,64,69^; (2) raclopride is a popular D_2_ tracer but has unreliable binding in the cortex and is, therefore, an inappropriate tracer to use for mapping D_2_ densities in the cortex, but we show its comparison to FLB457 and another D_2_ tracer, fallypride, for completeness^98,99,103^; and (3) the chosen carfentanil (MOR) map was collated across carfentanil images in the PET Turku Centre database—because our alternative map is a partly overlapping subset of participants, we did not combine the tracers into a single mean map^93,101^.

Synapse density in the cortex was measured in 76 healthy adults (45 males, 48.9 ± 18.4 years of age) by administering [^11^C]UCB-J, a PET tracer that binds to the synaptic vesicle glycoprotein 2A (SV2A)^104^. Data were collected on an HRRT PET camera for 90 minutes after injection. Non-displaceable binding potential (BP_ND_) was modeled using SRTM2, with the centrum semiovale as reference and $k^{\prime}$ fixed to 0.027 (population value). This group-averaged map was first presented in ref. ^105^.

### Autoradiography receptor data acquisition

Receptor autoradiography data were originally acquired as described in ref. ^6^. Fifteen neurotransmitter receptor densities across 44 cytoarchitectonically defined areas were collected in three postmortem brains (age range: 72–77 years, two males). See Supplementary Table 1 for a complete list of receptors included in the autoradiography dataset; see Supplementary Table 2 in ref. ^6^ for the originally reported receptor densities; and see https://github.com/AlGoulas/receptor_principles for machine-readable Python numpy files of receptor densities^37^. To best compare PET data analyses with the autoradiography dataset, a region-to-region mapping was manually created between the 44 available cortical areas in the autoradiography dataset and the 50 left hemisphere cortical Schaefer-100 regions. Four regions in the Schaefer atlas did not have a suitable mapping to the autoradiography atlas. As such, the 44-region autoradiography atlas was converted to 46 Schaefer left hemisphere regions. Finally, receptor densities were concatenated and z-scored to create a single map of receptor densities across the cortex.

### Structural and functional data acquisition

Following the procedure described in ref. ^106^, we obtained structural and functional MRI data for 326 unrelated participants (age range: 22–35 years, 145 males) from the HCP (S900 release^29^). All four resting-state functional MRI scans (two scans (R/L and L/R phase-encoding directions) on day 1 and two scans (R/L and L/R phase-encoding directions) on day 2, each about 15 minutes long; TR = 720 ms), as well as diffusion-weighted imaging (DWI) data were available for all participants. All the structural and functional MRI data were pre-processed using HCP minimal pre-processing pipelines^29,107^. We provide a brief description of data pre-processing below, whereas detailed information regarding data acquisition and pre-processing is available elsewhere^29,107^.

### Structural network reconstruction

DWI data were pre-processed using the MRtrix3 package^108^ (https://www.mrtrix.org/). More specifically, fiber orientation distributions were generated using the multi-shell, multi-tissue constrained spherical deconvolution algorithm from MRtrix^109,110^. White matter edges were then reconstructed using probabilistic streamline tractography based on the generated fiber orientation distributions^111^. The tract weights were then optimized by estimating an appropriate cross-section multiplier for each streamline following the procedure proposed by ref. ^112^, and a connectivity matrix was built for each participant using the 100-region Schaefer parcellation^12^. A group consensus binary network was constructed using a method that preserves the density and edge-length distributions of the individual connectomes^113^. Edges in the group consensus network were assigned weights by averaging the log-transformed streamline count of non-zero edges across participants. Edge weights were then scaled to values between 0 and 1.

### Functional network reconstruction

All 3T functional MRI time series were corrected for gradient non-linearity, head motion using a rigid body transformation and geometric distortions using scan pairs with opposite phase encoding directions (R/L and L/R)^106^. Further pre-processing steps include co-registration of the corrected images to the T1w structural MR images, brain extraction, normalization of whole brain intensity, high-pass filtering (>2,000s full width at half maximum (FWHM); to correct for scanner drifts) and removing additional noise using the ICA-FIX process^106,114^. The pre-processed time-series were then parcellated to 100 cortical brain regions according to the Schaefer atlas^12^. The parcellated time series were used to construct functional connectivity matrices as a Pearson correlation coefficient between pairs of regional time series for each of the four scans of each participant. A group-average functional connectivity matrix was constructed as the mean functional connectivity across all individuals and scans.

### Structure–function coupling

Structure–function coupling at every brain region is defined as the adjusted *R*^2^ of a simple linear regression model that fits regional communicability (that is, the communicability between a brain region to every other brain region) to regional functional connectivity (that is, the functional connectivity between a brain region and every other brain region). Communicability is defined as the weighted average of all walks and paths between two brain regions and represents diffusive communication^26,115^. Additionally, communicability has been previously demonstrated as an important bridge between brain structure and function^27^. In the receptor-informed model, receptor similarity between the region of interest and every other region was included as an additional independent variable. The significance of the receptor-informed structure–function coupling was assessed against a null distribution of adjusted *R*^2^ from a model that adds a rotated regional receptor similarity vector (10,000 repetitions). This ensures that the increase in *R*^2^ when receptor information is included in the model is robust against the addition of a random variable with identical spatial autocorrelation.

### MEG power

Six-minute resting-state eyes-open magenetoencephalography (MEG) time series were acquired from the HCP (S1200 release) for 33 unrelated participants (age range: 22–35 years, 17 males)^29,107^. Complete MEG acquisition protocols can be found in the HCP S1200 Release Manual. For each participant, we computed the power spectrum at the vertex level across six different frequency bands: delta (2–4 Hz), theta (5–7 Hz), alpha (8–12 Hz), beta (15–29 Hz), low gamma (30–59 Hz) and high gamma (60–90 Hz), using the open-source software Brainstorm^116^. The pre-processing was performed by applying notch filters at 60, 120, 180, 240 and 300 Hz and was followed by a high-pass filter at 0.3 Hz to remove slow-wave and DC-offset artifacts. Pre-processed sensor-level data were used to obtain a source estimation on HCPʼs fsLR4k cortex surface for each participant. Head models were computed using overlapping spheres, and the data and noise covariance matrices were estimated from the resting-state MEG and noise recordings. Brainstorm’s linearly constrained minimum variance (LCMV) beamformers method was applied to obtain the source activity for each participant. Welch’s method was then applied to estimate power spectrum density (PSD) for the source-level data, using overlapping windows of length 4 seconds with 50% overlap. Average power at each frequency band was then calculated for each vertex (that is, source). Source-level power data were then parcellated into 100 cortical regions for each frequency band^12^.

### ENIGMA cortical abnormality maps

The ENIGMA (Enhancing Neuroimaging Genetics through Meta-Analysis) consortium is a data-sharing initiative that relies on standardized image acquisition and processing pipelines, such that disorder maps are comparable^117^. Patterns of cortical abnormality were collected for 13 neurological, neurodevelopmental and psychiatric disorders from the ENIGMA consortium and the Enigma toolbox (https://github.com/MICA-MNI/ENIGMA; ref. ^118^), including: 22q11.2 deletion syndrome (22q)^119^, ADHD^120^, ASD^121^, idiopathic generalized epilepsy^122^, right temporal lobe epilepsy^122^, left temporal lobe epilepsy^122^, depression^123^, OCD^124^, schizophrenia^125^, BD^126^, obesity^127^, schizotypy^128^ and PD^129^. Although most disorders show decreases in cortical thickness, some (for example, 22q, ASD and schizotypy) also show regional increases in cortical thickness. We, therefore, refer to the disorder profiles as ‘cortical abnormalities’. All cortical abnormality maps were collected from adult patients (except for ASD for which only an age-aggregated (2–64 years) map was available), following identical processing protocols, for a total of over 21,000 scanned patients against almost 26,000 controls. The values for each map are z-scored effect sizes (Cohen’s *d*) of cortical thickness in patient populations versus healthy controls. Note that the native and only representatin of ENIGMA datasets is the Desikan–Killiany atlas (68 cortical regions)^130^. For visualization purposes, data are inverted such that larger values represent greater cortical thinning. Imaging and processing protocols can be found at http://enigma.ini.usc.edu/protocols/.

### Dominance analysis

Dominance analysis seeks to determine the relative contribution (‘dominance’ of each independent variable to the overall fit (adjusted *R*^2^)) of the multiple linear regression model (https://github.com/dominance-analysis/dominance-analysis (ref. ^31^)). This is done by fitting the same regression model on every combination of input variables (2^*p*^ − 1 submodels for a model with *p* input variables). Total dominance is defined as the average of the relative increase in *R*^2^ when adding a single input variable of interest to a submodel, across all 2^*p*^ − 1 submodels. The sum of the dominance of all input variables is equal to the total adjusted *R*^2^ of the complete model, making total dominance an intuitive method that partitions the total effect size across predictors. Therefore, unlike other methods of assessing predictor importance, such as methods based on regression coefficients or univariate correlations, dominance analysis accounts for predictor–predictor interactions and is interpretable. Dominance was then normalized by the total fit (${R}_{{{{\rm{adj}}}}}^{2}$) of the model, to make dominance fully comparable both within and across models.

### Cognitive meta-analytic activation

Probabilistic measures of the association between voxels and cognitive processes were obtained from Neurosynth, a meta-analytic tool that synthesizes results from more than 15,000 published functional MRI studies by searching for high-frequency keywords (such as ‘pain’ and ‘attention’) that are published alongside functional MRI voxel coordinates (https://github.com/neurosynth/neurosynth, using the volumetric association test maps^34^). This measure of association is the probability that a given cognitive process is reported in the study if there is activation observed at a given voxel. Although more than 1,000 cognitive processes are reported in Neurosynth, we focused primarily on cognitive function and, therefore, limit the terms of interest to cognitive and behavioral terms. These terms were selected from the Cognitive Atlas, a public ontology of cognitive science^131^, which includes a comprehensive list of neurocognitive processes. We used 123 terms, ranging from umbrella terms (‘attention’ and ‘emotion’) to specific cognitive processes (‘visual attention’ and ‘episodic memory’), behaviors (‘eating’ and ‘sleep’) and emotional states (‘fear’ and ‘anxiety’). The coordinates reported by Neurosynth were parcellated according to the Schaefer-100 atlas and z-scored^12^. The probabilistic measure reported by Neurosynth can be interpreted as a quantitative representation of how regional fluctuations in activity are related to psychological processes. The full list of cognitive processes is shown in Supplementary Table 2.

### Partial least squares analysis

Partial least squares (PLS) analysis was used to relate neurotransmitter receptor distributions to functional activation. PLS is an unsupervised multivariate statistical technique that decomposes the two datasets into orthogonal sets of latent variables with maximum covariance^132^. The latent variables consist of receptor weights, cognitive weights and a singular value that represents the covariance between receptor distributions and functional activations that is explained by the latent variable. Receptor and cognitive scores are computed by projecting the original data onto the respective weights, such that each brain region is assigned a receptor and cognitive score. Finally, receptor loadings are computed as the Pearson’s correlation between receptor densities and receptor scores and vice versa for cognitive loadings. Note that PLS analysis does not (1) speak to causal relationships between receptors and cognition, (2) make specific univariate receptor–cognition associations and (3) preclude the existence of additional relationships between receptors and cognitive function.

The significance of the latent variable was assessed on the singular value, against the spin-test (see the ‘Null models’ section). In the present report, only the first latent variable was significant; the remaining latent variables were not analyzed further. Finally, the correlation between receptor and cognitive scores was cross-validated (see the ‘Distance-dependent cross-validation’ section). The empirical correlation between receptor and cognitive scores across all brain regions was *r*(98) = 0.70; the mean training set correlation was *r*(98) = 0.71; and the mean test set correlation was *r*(98) = 0.54 and *P*_spin_ = 0.046, one-sided.

### Distance-dependent cross-validation

The robustness of each multilinear model was assessed by cross-validating the model by using a distance-dependent method^28^. Specifically, this method was applied to every multilinear regression model (Figs. 3c, 4 and 6) and the PLS model (Fig. 5). For each brain region (source node), we selected the 75% closest regions as the training set and the remaining 25% of brain regions as the test set, for a total of 100 repetitions in the Schaefer atlas and 68 repetitions in the Desikan–Killiany atlas. This stratification procedure minimizes the dependence among the two sets due to spatial autocorrelation. In the case of multilinear regression models, the model was fit on the training set, and the predicted test set output variable (regional functional connectivity, MEG power or disorder maps) was correlated to the empirical test set values. The distribution of Pearson’s correlations between predicted and empirical variables across all repetitions (that is, all brain regions) can be found in Supplementary Fig. 2 (structure–function coupling), Supplementary Fig. 3 (MEG power) and Supplementary Fig. 7 (disorder maps).

In the case of the PLS analysis, the model was fit on the training set, and the weights were projected onto the test set to calculate predicted receptor and cognitive scores. Training and test sets were defined as described above, and the procedure was repeated for each brain region as the source node (100 repetitions). The correlation between receptor and cognitive score was separately calculated in the training and test set. The significance of the mean out-of-sample correlation was assessed against a permuted null model, constructed by repeating the cross-validation on spatial autocorrelation-preserving permutations of the functional association matrix (1,000 repetitions; Fig. 5d).

### Null models

Spatial autocorrelation-preserving permutation tests were used to assess statistical significance of associations across brain regions, termed ‘spin tests’^24,25,133^. We created a surface-based representation of the parcellation on the FreeSurfer fsaverage surface via files from the Connectome Mapper toolkit (https://github.com/LTS5/cmp). We used the spherical projection of the fsaverage surface to define spatial coordinates for each parcel by selecting the coordinates of the vertex closest to the center of the mass of each parcel. These parcel coordinates were then randomly rotated, and original parcels were reassigned the value of the closest rotated parcel (10,000 repetitions). Parcels for which the medial wall was closest were assigned the value of the next most proximal parcel instead. The procedure was performed at the parcel resolution rather than the vertex resolution to avoid upsampling the data and to each hemisphere separately. Note that the spin test was not applied to autroadiography data because of missing samples. A permutation test was applied instead.

A second null model was used to test whether receptor similarity is greater in connected regions than unconnected regions. This model generates a null structural connectome that preserves the density, edge length and degree distributions of the empirical structural connectome^22,133^. In brief, edges were binned according to Euclidean distance. Within each bin, pairs of edges were selected at random and swapped. This procedure was then repeated 10,000 times. To compute a *P* value, the mean receptor similarity of unconnected edges was subtracted from the mean receptor similarity of connected edges, and this difference was compared to a null distribution of differences computed on the rewired networks.

### Reporting Summary

Further information on research design is available in the Nature Research Reporting Summary linked to this article.

## Online content

Any methods, additional references, Nature Research reporting summaries, source data, extended data, supplementary information, acknowledgements, peer review information; details of author contributions and competing interests; and statements of data and code availability are available at 10.1038/s41593-022-01186-3.

## Supplementary information

Supplementary Information Supplementary Figs. 1–13 and Supplementary Tables 1 and 2.

Reporting Summary

Supplementary Table 3 Methodological details for each PET tracer.

## Data Availability

All data used to perform the analyses can be found at https://github.com/netneurolab/hansen_receptors. Volumetric PET images, including receptor images and synaptic density, are included in neuromaps (https://github.com/netneurolab/neuromaps) where they can be converted between template spaces^94^. Autoradiography data are available in Supplementary Table 2 of ref. ^6^. The HCP dataset, including diffusion-weighted MRI, functional MRI and MEG, is available at https://db.humanconnectome.org/. Neurosynth data are available at https://neurosynth.org/. The ENIGMA datasets are available through the ENIGMA consortium and the ENIGMA Toolbox (https://github.com/MICA-MNI/ENIGMA (ref. ^134^)). Parcellation atlases, including the Schaefer-100 and Desikan–Killiany atlas, were obtained from netneurotools (https://github.com/netneurolab/netneurotools).

## Code availability

All code used to perform the analyses can be found at https://github.com/netneurolab/hansen_receptors.
