# Supplementary material for: Mapping neurotransmitter systems to the structural and functional organization of the human neocortex
Source: Nat Neurosci. 2022 Oct 27;25(11):1569–81. doi: 10.1038/s41593-022-01186-3 (PMC9630096; doi:10.1038/s41593-022-01186-3)
Supplement: Supplementary file 1 — Supplementary Figs. 1–13 and Supplementary Tables 1 and 2. [file 41593_2022_1186_MOESM1_ESM.pdf]

nature neuroscience

Resource https://doi.org/10.1038/s41593-022-01186-3

Mapping neurotransmitter systems to the structural and functional organization of the human neocortex

In the format provided by the authors and unedited

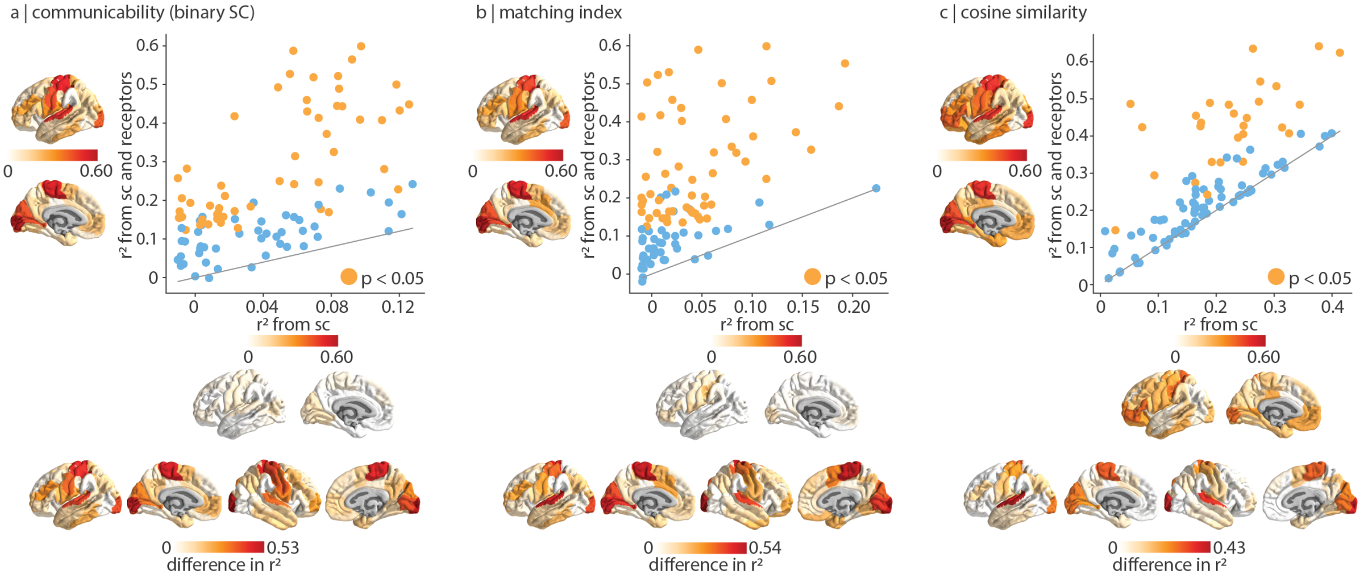

Figure S1. **Alternative representations of the structural connectome used in structure-function coupling analysis |** Structure-function coupling (corresponding to Fig. 3c) was recomputed using (a) communicability calculated on the binary connectome, (b) matching index computed on the weighted connectome, and (c) cosine similarity of the weighted connectome. Regional structure-function coupling was computed as the fit (R^2_{adj}) between a measure of the structural connectome and functional connectivity. Structure-function coupling at each brain region is plotted when receptor similarity is excluded (x-axis) and included (y-axis) in the model. Yellow points indicate brain regions where receptor information significantly augments structure-function coupling ($p_{\text{spin}} < 0.05$, FDR-corrected, one-sided).

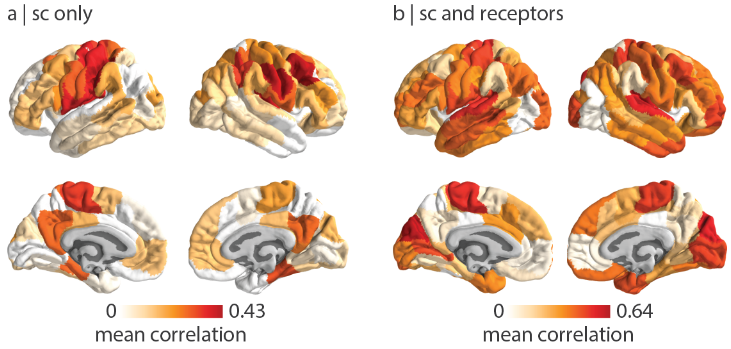

Figure S2. **Cross-validating structure-function coupling models |** At every brain region, distance-dependent cross-validation was applied to the (a) structure-function coupling model, and (b) the receptor-informed structure-function coupling model. The mean correlation (Pearson's r) between empirical and predicted values in the test set is shown on the brain surface.

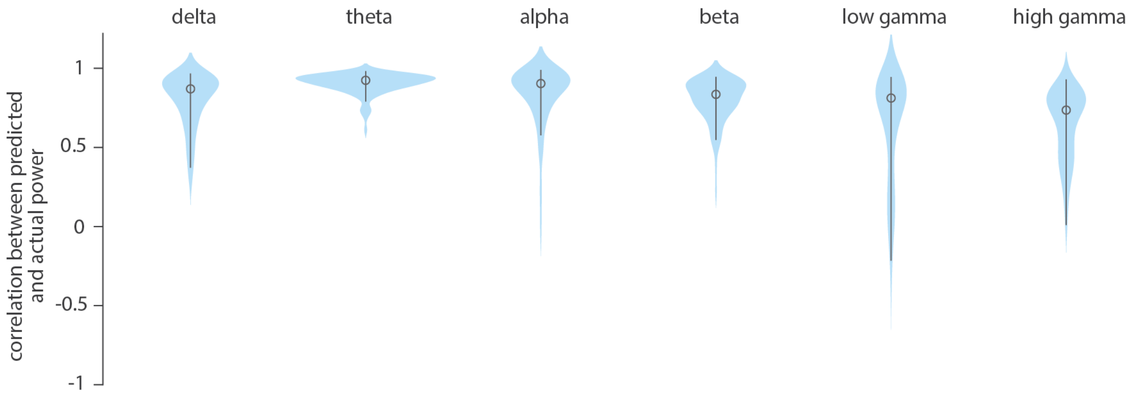

Figure S3. **Cross-validating models that predict MEG power distribution from receptor/transporter densities |** All six multilinear models between receptor/transporter densities and MEG power distributions were cross-validated using a distance-dependent method. This method selects the 25% of regions closest to a source-region as a training set and the remaining 75% of regions as the test set. The procedure is repeated for each brain region as the source region (100 iterations). We assessed the prediction by correlating predicted power to the empirical power in the test set. Circles in the violin plot represent the median and lines span the non-outliner minima and maxima of the distribution.

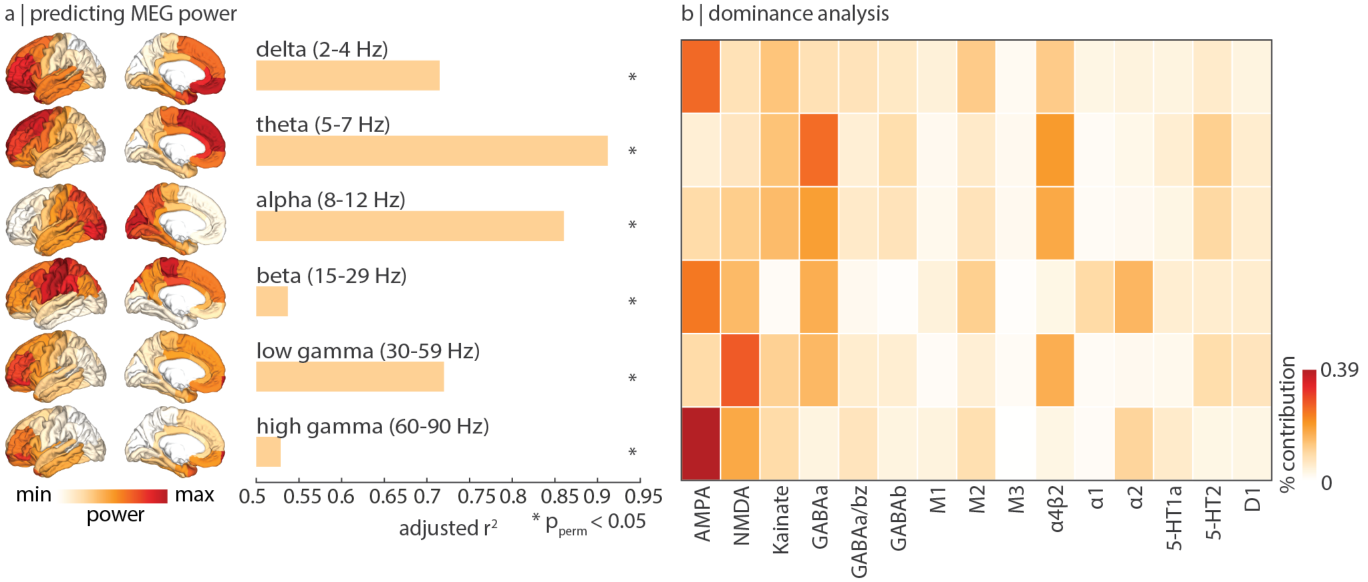

Figure S4. **Excitatory ionotropic receptor densities shape neural dynamics |** Multilinear regression models were fit between autoradiography-derived neurotransmitter receptor densities and MEG power, done analogously in Fig. 4. (a) Autoradiography-derived receptor densities map closely to neural dynamics. The significance of each model is assessed using a permutation test and is corrected for multiple comparisons (FDR). Asterisks denote significant models (FDR-corrected $p_{\text{perm}} < 0.05$). Delta $R^2_{\text{adj}}(30) = 0.71$, $p_{\text{spin}} = 0.0001$; theta $R^2_{\text{adj}}(30) = 0.91$, $p_{\text{spin}} = 0.0001$; alpha $R^2_{\text{adj}}(30) = 0.86$, $p_{\text{spin}} = 0.0001$; beta $R^2_{\text{adj}}(30) = 0.54$, $p_{\text{spin}} = 0.0002$; low gamma $R^2_{\text{adj}}(30) = 0.72$, $p_{\text{spin}} = 0.0001$; high gamma $R^2_{\text{adj}}(30) = 0.53$, $p_{\text{spin}} = 0.0003$. (b) Dominance analysis distributes the fit of the model across input variables such that the contribution of each variable can be assessed and compared to other input variables. The percent contribution of each input variable is defined as the variable's dominance normalized by the total fit (R^2_{adj}) of the model.

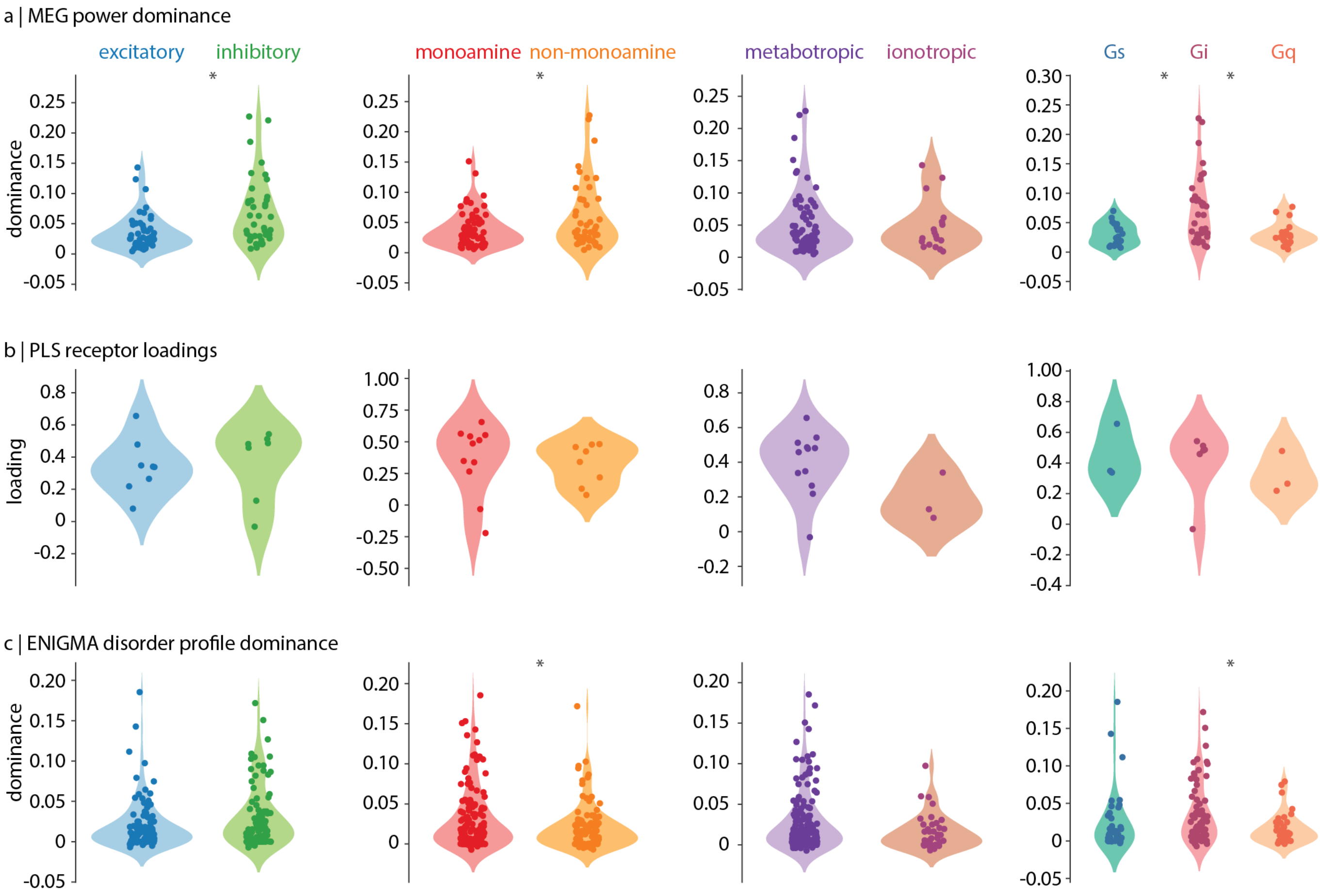

Figure S5. We compared the results from the MEG power dominance analysis (Fig. 4 in the main text), the PLS analysis with cognitive activations (Fig. 5 in the main text), and the disease profile dominance analysis (Fig. 6 in the main text) across different classes of receptors (excitatory vs. inhibitory, monoamine vs. non-monoamine, metabotropic vs. ionotropic, Gs- vs. Gi- vs. Gq-coupled pathways). Asterisks indicate significance ($p < 0.05$, Welch's t-test, two-sided). (a) Receptor dominance towards predicting MEG power is significantly greater in inhibitory versus excitatory receptors ($p = 0.001$), non-monoamine versus monoamine receptors ($p = 0.020$), Gi- versus Gs-coupled receptors ($p = 0.006$), and in Gi- versus Gq-coupled receptors ($p = 0.006$). (b) Receptor loadings do not show significant differences across the different receptor classes. (c) Receptor dominance towards predicting disorder profiles is significantly greater in monoamine versus non-monoamine receptors ($p = 0.025$) and in Gi- versus Gq-coupled receptors ($p = 0.018$).

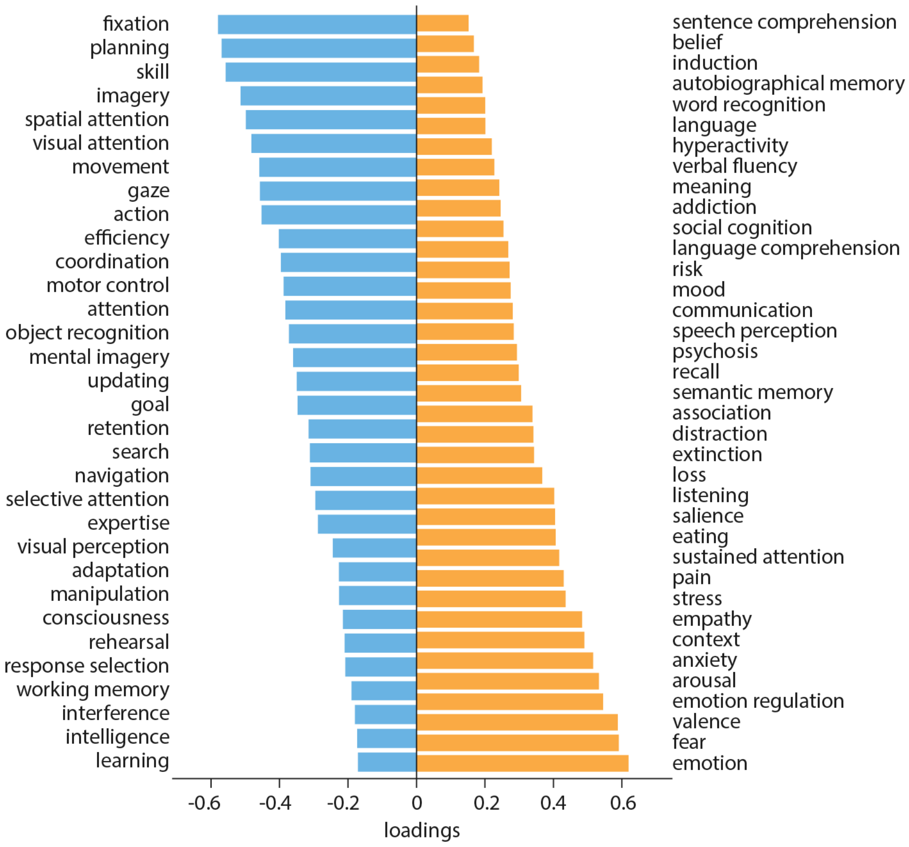

Figure S6. **Neurosynth cognitive loadings |** The loading for each cognitive process is calculated as the Pearson's correlation between functional activations across brain regions and PLS-derived receptor scores. We estimated bootstrap-estimated 95% confidence intervals (10 000 bootstrap samples) and do not show cognitive processes with a confidence interval that changes sign.

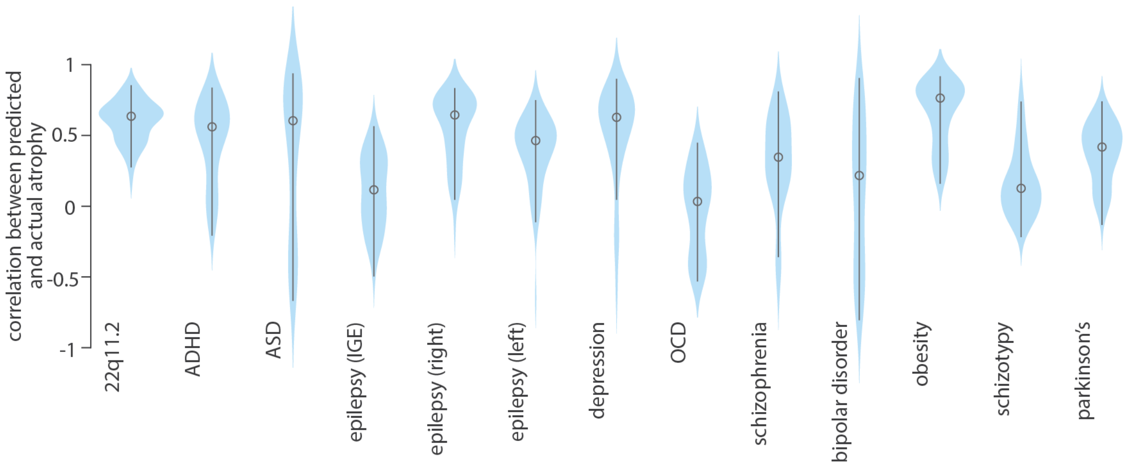

Figure S7. **Cross-validating models that predict disorder-specific cortical abnormality from receptor/transporter densities |** All thirteen multilinear models between receptor/transporter densities and disorder-specific cortical abnormality were cross-validated using a distance-dependent method. This method selects the 25% of regions closest to a source-region as a training set and the remaining 75% of regions as the test set. The procedure is repeated for each brain region as the source region (68 iterations). We assessed the prediction by correlating predicted atrophy to the empirical atrophy in the test set. Note that this analysis is conducted using the Desikan-Killiany atlas because this is the only representation of ENIGMA datasets. Circles in the violin plot represent the median and lines span the non-outliner minima and maxima of the distribution.

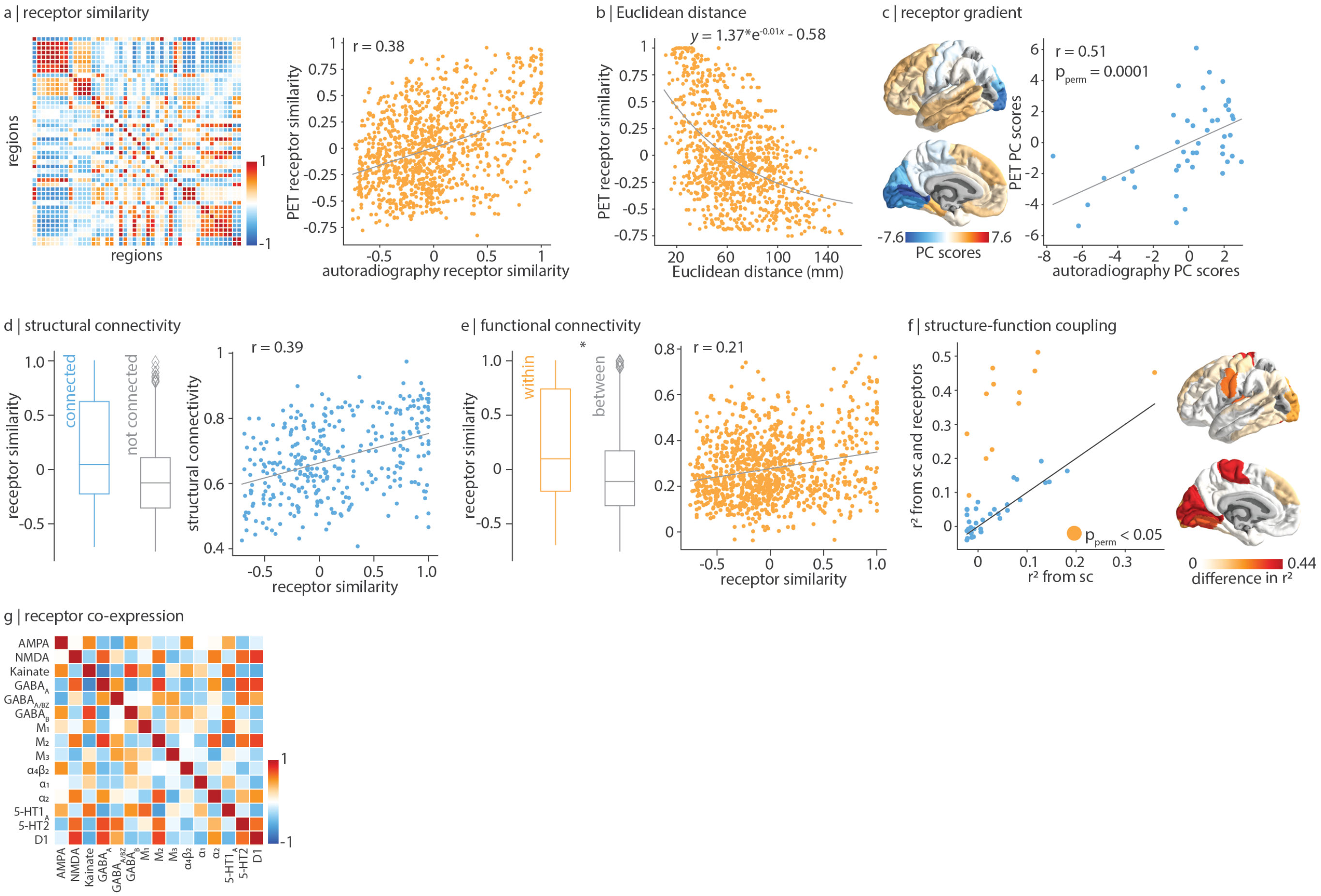

Figure S8. **Autoradiography-informed neurotransmitter receptor densities follow similar organizational principles as PET-informed neurotransmitter receptor densities |** Autoradiography images of fifteen neurotransmitter receptors across three post-mortem brains were acquired by [6]. (a) The receptor similarity matrix is constructed by correlating receptor fingerprints at each pair of brain regions (left). PET-derived receptor similarity is correlated to autoradiography-derived receptor similarity (Pearson's $r(1033) = 0.38$, $p = 6.7 \times 10^{-38}$, CI $= [0.33, 0.44]$, two-sided; right). (b) Receptor similarity decays exponentially with Euclidean distance. (c) The first principal component of autoradiography-derived receptor density (left brain plot) is non-significantly correlated with the first principal component of PET-derived receptor density (Pearson's $r(44) = 0.51$, $p_{\text{perm}} = 0.0001$, CI $= [0.26, 0.70]$ two-sided). (d) Receptor similarity is non-significantly greater between pairs of regions that are physically connected, against a degree- and edge-length-preserving null model (left; $p = 0.19$, two-sided, $N_{\text{connected}} = 331$ edges, $N_{\text{not connected}} = 704$ edges [22]), and is significantly correlated with structural connectivity (right; Pearson's $r(329) = 0.39$, $p = 1.4 \times 10^{-13}$, CI $= [0.30, 0.48]$, two-sided). (e) Receptor similarity is significantly greater in regions within the same functional network as opposed to between functional networks (left; $p_{\text{spin}} = 0.03$, two-sided, $N_{\text{within}} = 161$ edges, $N_{\text{between}} = 874$ edges), and is correlated to functional connectivity (right; Pearson's $r(1033) = 0.21$, $p = 1.1 \times 10^{-12}$, CI $= [0.16, 0.28]$, two-sided). (f) Consistent with PET-derived results, receptor similarity augments structure-function coupling in visual, paracentral, and somatomotor regions. Yellow points indicate brain regions where structure-function coupling (R^2_{adj}) is significantly greater when receptor similarity is included in the model ($p_{\text{perm}} < 0.05$, FDR-corrected, one-sided). (g) Receptor co-expression (Pearson's correlation) for every pair of receptors across 46 brain regions. Asterisks in panel (e) denote significance. Boxplots in (d) and (e) represent the 1st, 2nd (median) and 3rd quartiles, whiskers represent the non-outlier end-points of the distribution, and diamonds represent outliers.

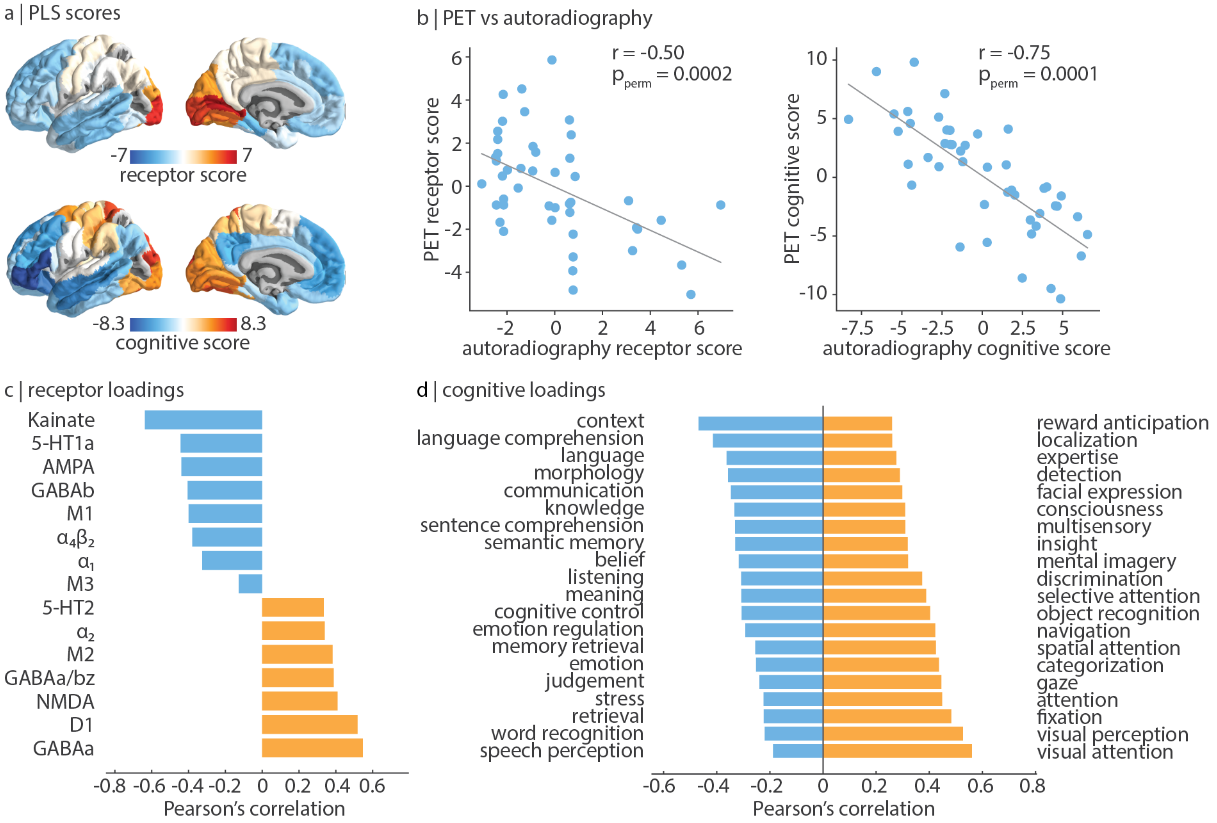

Figure S9. **Mapping autoradiography-derived receptors to cognition** | Partial least squares analysis was applied to autoradiography-derived receptor densities and Neurosynth-derived cognitive functional activations, done analogously in Fig. 5. (a) Receptor (top) and cognitive (bottom) score patterns follow a similar sensory-fugal gradient. (b) Autoradiography-derived PLS scores are correlated with PET-derived PLS scores. (c) Receptor loadings are defined as the Pearson's correlation between each receptor's distribution across the cortex and the PLS-derived receptor scores and can be interpreted as the contribution of each receptor to the latent variable. (c) Cognitive loadings are shown for all stable positively- and negatively-loaded cognitive processes.

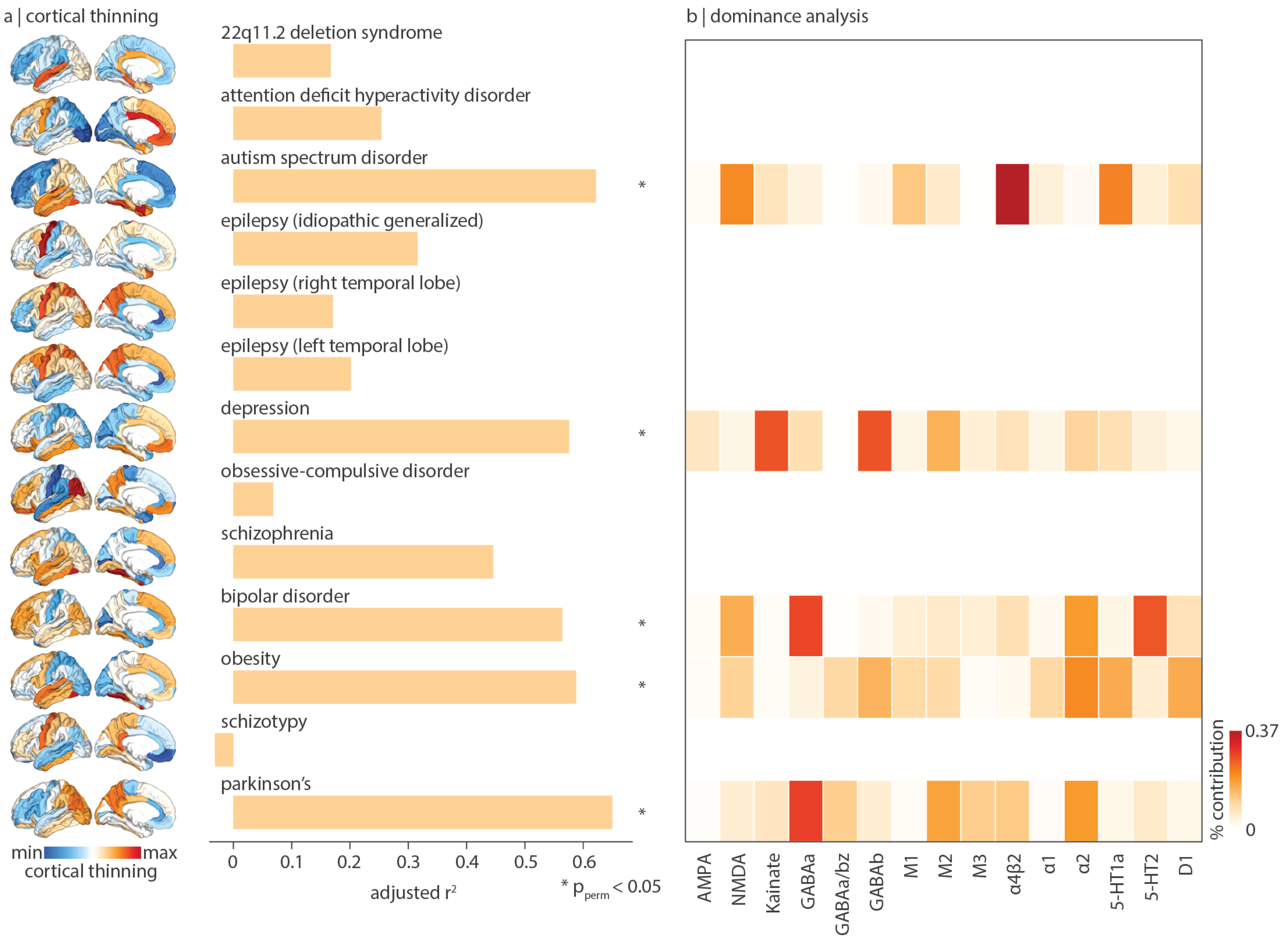

Figure S10. **Mapping autoradiography-derived receptors to disease vulnerability** | For each disorder, we fit a multilinear regression model between autoradiography-derived receptor densities and cortical abnormality, done analogously in Fig. 6. (a) Model fit (adjusted R^2) varies across disorders. The significance of each model is assessed using a permutation test and is corrected for multiple comparisons (FDR). Asterisks denote significant models (FDR-corrected $p_{\text{perm}} < 0.05$). 22q11.2 deletion $R^2_{\text{adj}}(17) = 0.17$, $p_{\text{spin}} = 0.28$; ADHD $R^2_{\text{adj}}(17) = 0.25$, $p_{\text{spin}} = 0.25$; autism $R^2_{\text{adj}}(17) = 0.62$, $p_{\text{spin}} = 0.01$; epilepsy (IGE) $R^2_{\text{adj}}(17) = 0.32$, $p_{\text{spin}} = 0.19$; epilepsy (right) $R^2_{\text{adj}}(17) = 0.17$, $p_{\text{spin}} = 0.28$; epilepsy (left) $R^2_{\text{adj}}(17) = 0.20$, $p_{\text{spin}} = 0.28$; depression $R^2_{\text{adj}}(17) = 0.58$, $p_{\text{spin}} = 0.01$; OCD $R^2_{\text{adj}}(17) = 0.07$, $p_{\text{spin}} = 0.41$; schizophrenia $R^2_{\text{adj}}(17) = 0.45$, $p_{\text{spin}} = 0.05$; bipolar $R^2_{\text{adj}}(17) = 0.56$, $p_{\text{spin}} = 0.01$; obesity $R^2_{\text{adj}}(17) = 0.59$, $p_{\text{spin}} = 0.01$; schizotypy $R^2_{\text{adj}}(17) = -0.03$, $p_{\text{spin}} = 0.55$; parkinson's $R^2_{\text{adj}}(17) = 0.65$, $p_{\text{spin}} = 0.01$. (b) Dominance analysis distributes the fit of the model across input variables such that the contribution of each variable can be assessed and compared to other input variables. The percent contribution of each input variable is defined as the variable's dominance normalized by the total fit (R^2_{adj}) of the model. Note that this analysis is conducted using the Desikan-Killiany atlas because this is the only representation of ENIGMA datasets [130].

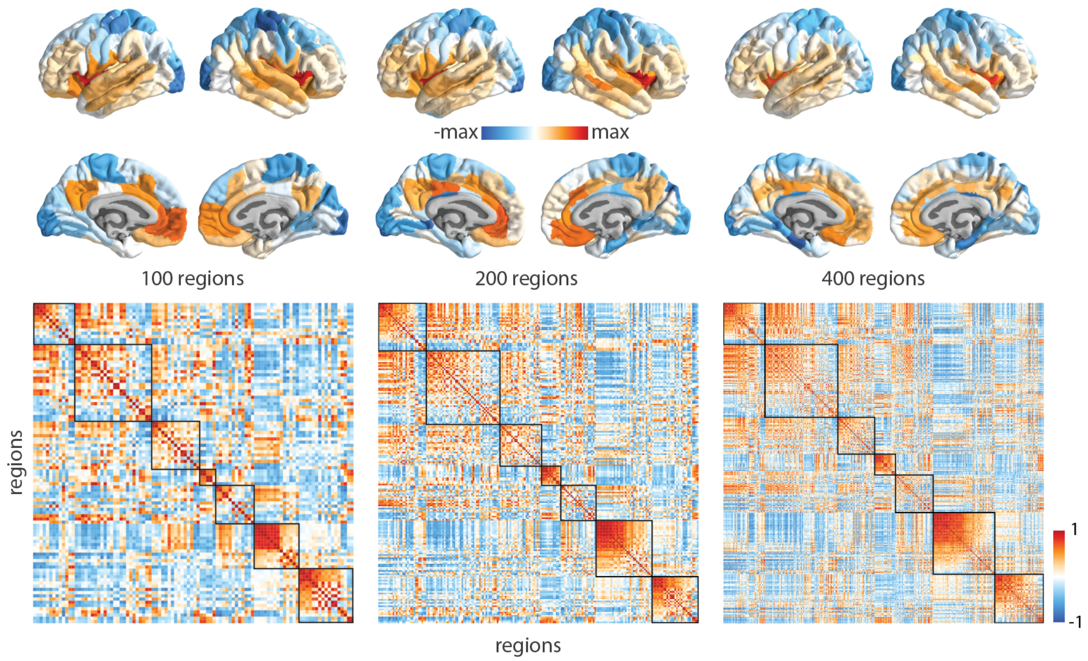

Figure S11. **Replicating results using different parcellation resolutions |** Top: first principal gradient of normalized neurotransmitter receptor/transporter density is consistent across three increasingly fine parcellation resolutions (100 regions (original), 200 regions, and 400 regions) [12]. Bottom: receptor similarity matrices also demonstrate high conformity across parcellation resolutions. Receptor similarity matrices are ordered by Yeo-Krienen intrinsic networks (order: frontoparietal, default mode, dorsal attention, limbic, ventral attention, somatomotor, visual) [23].

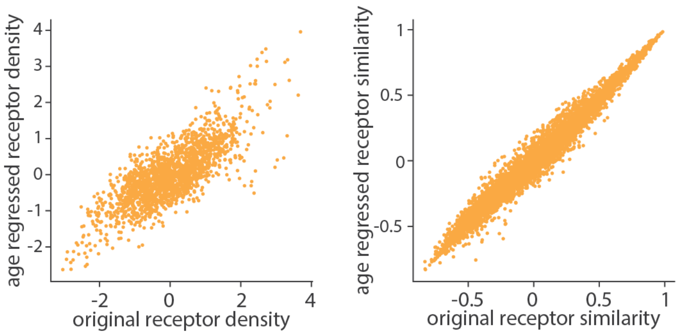

Figure S12. **Age has negligible effect on the reported findings |** To test age effects of the PET tracer images, we regressed out the relationship between mean age of each tracer map and z-scored receptor densities, at each brain region separately. Age has little impact on receptor density (left; Pearson's $r(4948) = 0.78$) and receptor similarity (right; Pearson's $r(4948) = 0.98$).

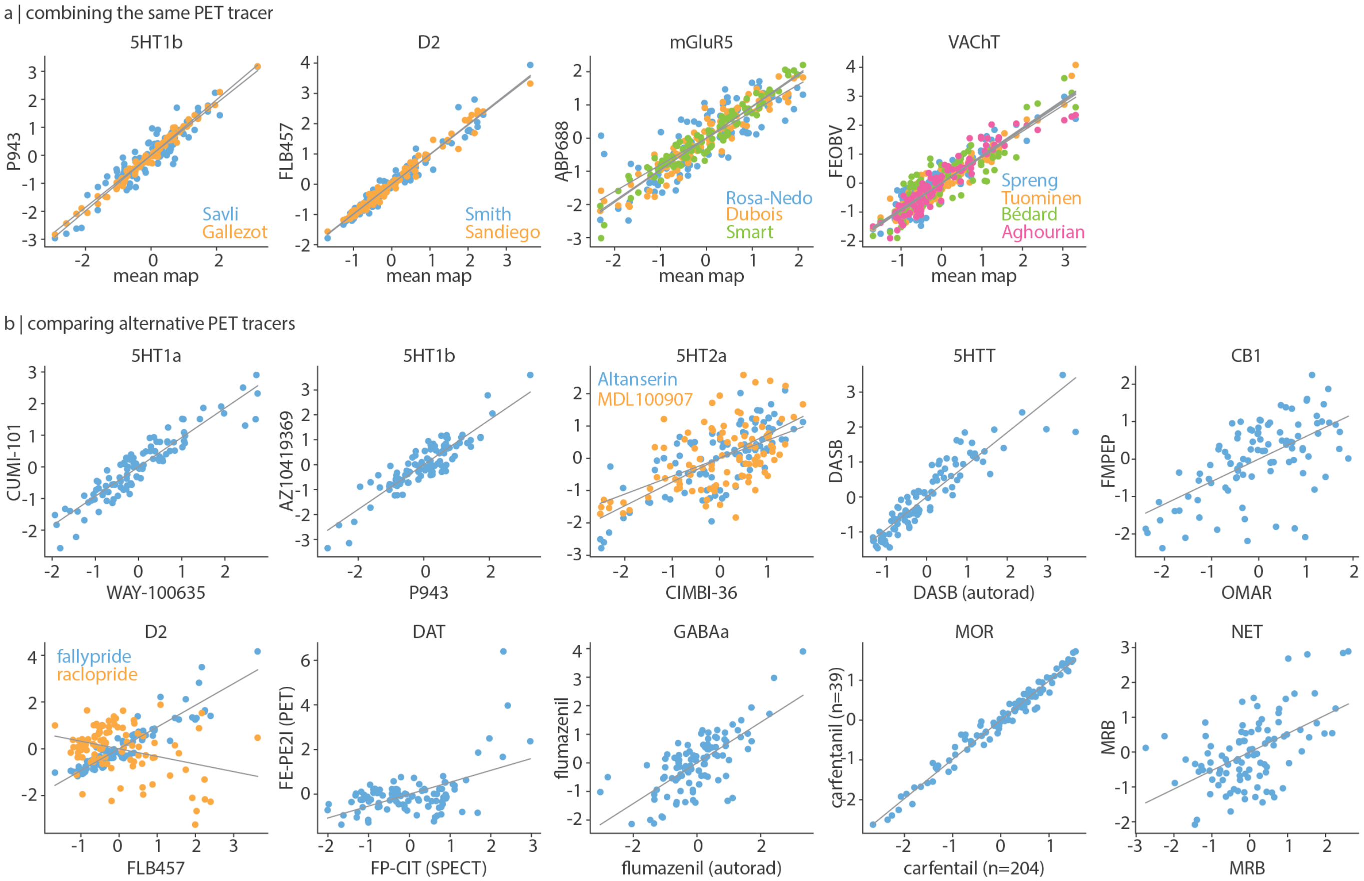

Figure S13. **Comparing different PET tracer images |** (a) PET maps of the same tracer were combined into a single average receptor/transporter map. Each individual PET tracer map (y-axis) is highly correlated to the mean map (x-axis). Names indicate the source of each PET map; see Table 1. (b) Multiple PET tracers were available for certain receptors/transporters. Scatter plots show the correlation between the selected tracer map (x-axis) and alternative maps (y-axis).

Receptor	Neurotransmitter	Excitatory/Inhibitory	Ionotropic/Metabotropic
AMPA	glutamate	excitatory	ionotropic
NMDA	glutamate	excitatory	ionotropic
Kainate	glutamate	excitatory	ionotropic
$GABA_A$	GABA	inhibitory	ionotropic
$GABA_{A/BZ}$	GABA	inhibitory	ionotropic
$GABA_B$	GABA	inhibitory	metabotropic
M_1	acetylcholine	excitatory	metabotropic
M_2	acetylcholine	inhibitory	metabotropic
M_3	acetylcholine	excitatory	metabotropic
$\alpha_4\beta_2$	acetylcholine	excitatory	ionotropic
α_1	norepinephrine	excitatory	metabotropic
α_2	norepinephrine	inhibitory	metabotropic
5-HT_{1A}	serotonin	inhibitory	metabotropic
5-HT_2	serotonin	excitatory	metabotropic
D_1	dopamine	excitatory	metabotropic

TABLE S1. **Neurotransmitter receptors included in the autoradiography dataset**

action	eating	insight	naming	semantic memory
adaptation	efficiency	integration	navigation	sentence comprehension
addiction	effort	intelligence	object recognition	skill
anticipation	emotion	intention	pain	sleep
anxiety	emotion regulation	interference	perception	social cognition
arousal	empathy	judgment	planning	spatial attention
association	encoding	knowledge	priming	speech perception
attention	episodic memory	language	psychosis	speech production
autobiographical memory	expectancy	language comprehension	reading	strategy
balance	expertise	learning	reasoning	strength
belief	extinction	listening	recall	stress
categorization	face recognition	localization	recognition	sustained attention
cognitive control	facial expression	loss	rehearsal	task difficulty
communication	familiarity	maintenance	reinforcement learning	thought
competition	fear	manipulation	response inhibition	uncertainty
concept	fixation	meaning	response selection	updating
consciousness	focus	memory	retention	utility
consolidation	gaze	memory retrieval	retrieval	valence
context	goal	mental imagery	reward anticipation	verbal fluency
coordination	hyperactivity	monitoring	rhythm	visual attention
decision	imagery	mood	risk	visual perception
decision making	impulsivity	morphology	rule	word recognition
detection	induction	motor control	salience	working memory
discrimination	inference	movement	search	
distraction	inhibition	multisensory	selective attention	

TABLE S2. **Neurosynth terms |** Terms that overlapped between the Neurosynth database [34] and the Cognitive Atlas [131] were included in the PLS analysis.
