# Supplementary material for: Mapping neurotransmitter systems to the structural and functional organization of the human neocortex
Source: Nat Neurosci. 2022 Oct 27;25(11):1569–81. doi: 10.1038/s41593-022-01186-3 (PMC9630096; doi:10.1038/s41593-022-01186-3)
Supplement: Supplementary file 2 — Reporting Summary [file 41593_2022_1186_MOESM2_ESM.pdf]

nature portfolio

Corresponding author(s): Bratislav Misic
Last updated by author(s): Aug 26, 2022

Reporting Summary

Nature Portfolio wishes to improve the reproducibility of the work that we publish. This form provides structure for consistency and transparency in reporting. For further information on Nature Portfolio policies, see our Editorial Policies and the Editorial Policy Checklist.

Statistics

For all statistical analyses, confirm that the following items are present in the figure legend, table legend, main text, or Methods section.

n/a	Confirmed	
☐	☒	The exact sample size (*n*) for each experimental group/condition, given as a discrete number and unit of measurement
☐	☒	A statement on whether measurements were taken from distinct samples or whether the same sample was measured repeatedly
☐	☒	The statistical test(s) used AND whether they are one- or two-sided<br>*Only common tests should be described solely by name; describe more complex techniques in the Methods section.*
☐	☒	A description of all covariates tested
☐	☒	A description of any assumptions or corrections, such as tests of normality and adjustment for multiple comparisons
☐	☒	A full description of the statistical parameters including central tendency (e.g. means) or other basic estimates (e.g. regression coefficient) AND variation (e.g. standard deviation) or associated estimates of uncertainty (e.g. confidence intervals)
☐	☒	For null hypothesis testing, the test statistic (e.g. *F*, *t*, *r*) with confidence intervals, effect sizes, degrees of freedom and *P* value noted<br>*Give P values as exact values whenever suitable.*
☒	☐	For Bayesian analysis, information on the choice of priors and Markov chain Monte Carlo settings
☒	☐	For hierarchical and complex designs, identification of the appropriate level for tests and full reporting of outcomes
☐	☒	Estimates of effect sizes (e.g. Cohen's *d*, Pearson's *r*), indicating how they were calculated

Our web collection on statistics for biologists contains articles on many of the points above.

Software and code

Policy information about availability of computer code

Data collection	enigmatoolbox v1.1.1 (https://github.com/MICA-MNI/ENIGMA) was used for fetching ENIGMA data.<br>MEG data was processed using the open software toolbox Brainstorm v220420.<br>HCP structural data was processed using MRtrix3 v3.0.0
Data analysis	All code used to analyze data can be found at https://github.com/netneurolab/hansen_receptors. Data was analyzed using Python 3.8.10, MATLAB R2022a, netneurotools v0.2.3 and neuromaps v0.0.3.

For manuscripts utilizing custom algorithms or software that are central to the research but not yet described in published literature, software must be made available to editors and reviewers. We strongly encourage code deposition in a community repository (e.g. GitHub). See the Nature Portfolio guidelines for submitting code & software for further information.

Data

Policy information about availability of data

All manuscripts must include a data availability statement. This statement should provide the following information, where applicable:

- Accession codes, unique identifiers, or web links for publicly available datasets
- A description of any restrictions on data availability
- For clinical datasets or third party data, please ensure that the statement adheres to our policy

All data used to perform the analyses can be found at https://github.com/netneurolab/hansen_receptors. Volumetric PET images, including receptor images and synaptic density, are included in neuromaps (https://github.com/netneurolab/neuromaps) where they can be easily converted between template spaces. Autoradiography data is available in Supplementary Table 2 of Zilles & Palomero-Gallagher 2017, Frontiers in Neuroanatomy. The HCP dataset, including diffusion weighted MRI, fMRI, and MEG is available at https://db.humanconnectome.org/. Neurosynth data is available at https://neurosynth.org/. The ENIGMA datasets are

available through the ENIGMA consortium and the ENIGMA toolbox (https://github.com/MICA-MNI/ENIGMA). Parcellation atlases including the Schaefer-100 and Desikan-Killiany atlas were fetched from netneurotools (https://github.com/netneurolab/netneurotools).

Field-specific reporting

Please select the one below that is the best fit for your research. If you are not sure, read the appropriate sections before making your selection.

☒ Life sciences ☐ Behavioural & social sciences ☐ Ecological, evolutionary & environmental sciences

For a reference copy of the document with all sections, see nature.com/documents/nr-reporting-summary-flat.pdf

Life sciences study design

All studies must disclose on these points even when the disclosure is negative.

Sample size	No sample size calculations were performed. We collated as many PET tracer images as possible to construct a comprehensive (19-receptor/transporter) atlas. HCP data was used because of the large number of subjects and the relatively equal male/female balance. For HCP data, only unrelated subjects were included. For PET data, only healthy subjects were included. The ENIGMA dataset was selected because of the large number of subjects in each meta-analysis and because it contains many brain maps for diseases/disorders/conditions that have been similarly processed such that comparison between datasets is possible.
Data exclusions	No data was excluded.
Replication	The analysis pipeline was conducted and replicated: (1) at the Schaefer-100 parcellation resolution, (2) at the Schaefer-200 parcellation resolution, (3) at the Schaefer-400 parcellation resolution, (4) using the 68-node Desikan Killiany atlas alongside structural/functional connectomes from the Lausanne atlas. Furthermore, we recalculated the receptor similarity matrix in a leave-one-out fashion, and confirmed that no single receptor/transporter exerts undue influence on this similarity matrix (correlation between leave-one-out similarity matrix and original similarity matrix >0.98 for all receptors). Finally, analyses were repeated using autoradiography data for 15 receptors as opposed to PET data for 19 receptors/transporters.
Randomization	No randomization was performed as this study does not include experimental groups.
Blinding	Blinding is not relevant to this study because it does not include experimental groups.

Reporting for specific materials, systems and methods

We require information from authors about some types of materials, experimental systems and methods used in many studies. Here, indicate whether each material, system or method listed is relevant to your study. If you are not sure if a list item applies to your research, read the appropriate section before selecting a response.

Materials & experimental systems

n/a	Involved in the study	
☒	☐	Antibodies
☒	☐	Eukaryotic cell lines
☒	☐	Palaeontology and archaeology
☒	☐	Animals and other organisms
☐	☒	Human research participants
☒	☐	Clinical data
☒	☐	Dual use research of concern

Methods

n/a	Involved in the study	
☒	☐	ChIP-seq
☒	☐	Flow cytometry
☐	☒	MRI-based neuroimaging

Human research participants

Policy information about studies involving human research participants

Population characteristics	Demographic information for all PET subjects can be found in Table 1.
Recruitment	Only data from healthy control subjects were used in the analyses.
Ethics oversight	Each individual PET study was approved, details can be found in the references found in Table 1.

Note that full information on the approval of the study protocol must also be provided in the manuscript.

Magnetic resonance imaging

Experimental design

Design type: Resting-state fMRI and diffusion-weighted MRI

Design specifications: Following the procedure described in Vos De Wael et al., 2018, we obtained structural and functional magnetic resonance imaging (MRI) data for 326 unrelated participants (age range 22–35 years, 145 males) from the Human Connectome Project (HCP; S900 release). All four resting state fMRI scans (two scans (R/L and L/R phase encoding directions) on day 1 and two scans (R/L and L/R phase encoding directions) on day 2, each about 15 min long; TR=720 ms), as well as diffusion weighted imaging (DWI) data were available for all participants. All the structural and functional MRI data were pre-processed using HCP minimal pre-processing pipelines. Detailed information regarding data acquisition and pre-processing is available elsewhere (Van Essen et al., 2013, Glasser et al., 2013)

Behavioral performance measures: No behavioural measures were recorded during the fMRI runs.

Acquisition

Imaging type(s): Functional and diffusion-weighted MRI

Field strength: 3T

Sequence & imaging parameters: Multi-band sequence; functional images have a 2-mm isotropic signal resolution, structural modalities were acquired on a Siemens Skyra 3T scanner and included a T1-weighted MPRAGE sequence at an isotropic resolution of 0.7mm, and a T2-weighted SPACE at an isotropic resolution of 0.7mm. More details on imaging protocols and procedures are available at http://protocols.humanconnectome.org/HCP/3T/imaging-protocols.html.

Area of acquisition: Whole-brain

Diffusion MRI ☐ Used ☐ Not used

Preprocessing

Preprocessing software: We used the HCP data that was previously preprocessed. This preprocessing was done using FSL 5.0.6, FreeSurfer 5.3.0-HCP, and Connectome Workbench v1.1.1.

Normalization: Image processing includes correcting for gradient distortion caused by non-linearities, correcting for bias field distortions, and registering the images to a standard reference space.

Normalization template: fs_LR_32k surface mesh

Noise and artifact removal: FMRIB's ICA-based X-noisefier (FIX) and global signal regression

Volume censoring: No volume censoring was performed.

Statistical modeling & inference

Model type and settings: Functional and structural connectomes were used for comparison with PET-derived receptor similarity.

Effect(s) tested: We tested whether receptor similarity is greater when regions are connected (SC) or within the same intrinsic functional network (fMRI).

Specify type of analysis: ☒ Whole brain ☐ ROI-based ☐ Both

Statistic type for inference (See Eklund et al. 2016): NA

Correction: NA

Models & analysis

n/a	Involved in the study
☐	☒ Functional and/or effective connectivity
☐	☒ Graph analysis
☐	☒ Multivariate modeling or predictive analysis

Functional and/or effective connectivity: We used functional connectivity, which was constructed by correlated pairwise regional functional time series, and averaging this across subjects.

Graph analysis: We used structural connectivity (weighted) matrices. Structural connectivity between pairs of regions was

Graph analysis	measured in terms of fiber density, defined as the number of streamlines between two regions, normalized by the average length of the streamlines and average surface area of the two regions. The goal of this normalization is to compensate for the bias toward longer fibers inherent in the tractography procedure, as well as differences in region size.
Multivariate modeling and predictive analysis	Regional vectors of functional and connectivity were used in a multilinear regression model which fit measures of structure (distance, path length, communicability) and receptor similarity to functional connectivity.
